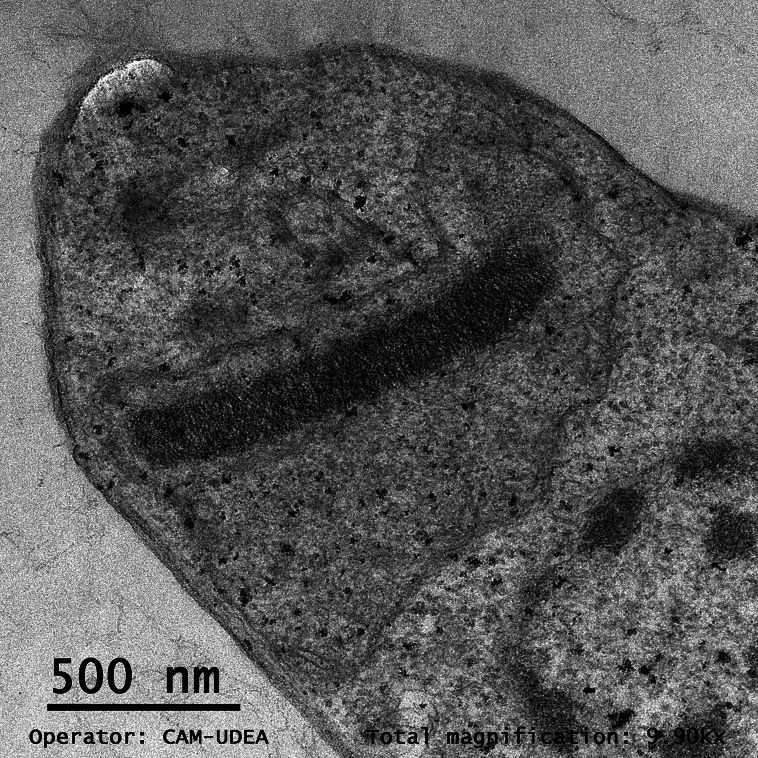# PUF3 RNA binding protein of *Trypanosoma cruzi* regulates mitochondrial morphology and function

**DOI:** 10.1016/j.heliyon.2024.e32810

**Published:** 2024-06-14

**Authors:** Ana María Mejía-Jaramillo, Geysson Javier Fernandez, Hader Ospina-Zapata, Ana Milena Murillo, Dianny Elizabeth Jimenez, Luis A. Gómez, Omar Triana-Chávez

**Affiliations:** a Biología y Control de Enfermedades Infecciosas, BCEI, Universidad de Antioquia, Medellín, Colombia; b Área de Ciencias Fundamentales, Universidad Eafit, Medellín, Colombia

**Keywords:** PUF3, RNA binding protein, *Trypanosoma cruzi*, Mitochondrial

## Abstract

The RNA-binding PUF proteins are post-transcriptional regulators found throughout the eukaryotic domain. In *Trypanosoma cruzi,* ten *Puf* genes termed *Puf*1 to *Puf*10 have been identified. Considering that the control of gene expression in this parasite is mainly at the post-transcriptional level, we characterized the PUF3 protein by knocking out and overexpressing the gene in *T. cruzi* epimastigotes and studied different genetic and biological features. The RNA-seq analyses in both genotypes showed significant changes in the number of regulated transcripts compared with wild-type parasites. Thus, the number of differentially expressed genes in the knockout (Δ*TcPuf3)* and the overexpressor (pTEX*TcPuf3)* were 238 and 187, respectively. In the knockout, a more significant proportion of genes was negatively regulated (166 out of 238). In contrast, in the overexpressor, positively regulated genes were predominant (149 out of 170). Additionally, when we predicted the subcellular location of the differentially expressed genes, the results revealed an important representation of nuclear genes encoding mitochondrial proteins. Therefore, we determined whether overexpression or knockout of *TcPuf3* could lead to changes in both mitochondrial structure and cellular respiration. When mitochondria from Δ*TcPuf3* and pTEX*TcPuf3* parasites were analyzed by transmission electron microscopy (TEM), it was observed that the overexpressor had an abnormal mitochondrial morphology, evidenced by swelling. The results associated with cellular respiration showed that both the Δ*TcPuf3* and pTEX*TcPuf3* had a lower efficiency in routine respiration and the electron transport system capacity. Likewise, the mitochondria from overexpressing parasites showed a slight hyperpolarization. Additionally, several biological features, depending on the function of the mitochondria, were altered, such as growth, cell division, metacyclogenesis, ROS production, and response to benznidazole. In conclusion, our results suggest that although PUF3 is not an essential protein in *T. cruzi*, it influences mitochondrial transcripts, affecting mitochondrial morphology and function.

## 1 Introduction

The PUF family RNA-binding proteins are post-transcriptional regulators throughout the eukaryotic domain. PUFs control the stability and translation of transcripts by binding to specific recognition sequences in the 3′untranslated regions (3′-UTRs) of mRNAs [[1], [2], [3]]. All PUFs contain a PUM-HD-type RNA-binding domain [4,5], which folds into an arc-like shape and can contact RNA and protein partners [6]. The established role of PUFs in eukaryotes is modulating the stability of RNA [3,7,8]. In addition to this well-known role, new findings in several organisms indicate that they can contribute to the translation of mRNA [3,9,10] and targeting of mRNAs to specific subcellular locations to provide spatial control of expression [3,11,12].

PUF proteins were first discovered as regulators of differentiation in *Drosophila melanogaster* [13,14] and subsequently have been found to have the same function in other animal species [15]. Most PUFs studied so far are cytosolic, but some are found in the nucleolus and are involved in rRNA processing [16,17]. Several structural and functional attributes of the *Puf* gene family have been widely studied in diverse groups such as plants [18], parasites [19] as well as in model organisms such as *D. melanogaster* [13,14], *Caenorhabditis elegans* [15,20] and *Saccharomyces cerevisiae* [21]. The cellular functions of PUF proteins involve (i) several *Puf* genes and (ii) different PUM-HD domains. Thus, whereas in plants between 19 and 26 Puf-like genes have been described [18], *S. cerevisiae* has only six cytosolic PUF proteins, none of which is essential, but cells lacking these proteins have extensive changes in gene expression [3].

The *Puf* gene family was previously identified in the genomes of TriTryp parasites such as *Leishmania major, Trypanosoma brucei,* and *T. cruzi*. In these parasites, 10 *Puf* genes termed *Puf1* to *Puf10* were identified, with the absence of *Puf4* in *L. major* [22]. In *T. cruzi, in silico* analyses predicted the existence of at least three major groups, based on their putative binding specificity, named NRE (for the *D. melanogaster* PUF binding element), UGUR (for the conserved core binding sequence in NRE) and Unknown (for unknown binding specificity). *TcPuf1* and *TcPuf2* belong to Group 1, *TcPuf3-6* and *TcPuf9* to Group 2, and finally, *TcPuf7-8* and *TcPuf10* to Group 3 [22].

Functional description and structural characteristics of *Puf* genes in trypanosomes have been primarily described in *T. brucei* [10,16,23]*.* Recently, the PUF3 protein was evaluated in this parasite; the study concluded that PUF3 may have a role in fine-tuning gene expression since RNA-interference PUF3 bloodstream forms had only minor growth defects, and the transcriptome was unaffected. Furthermore, procyclic forms without PUF3 grew slower, but ectopic expression of PUF3 impaired their viability, suggesting a toxic effect of the protein [24].

In *T. cruzi,* the only PUF proteins functionally characterized so far are PUF1 and PUF6, suggesting that these proteins regulate the half-lives of their associated transcripts via differential association with mRNA degradation complexes throughout its life cycle [22,25,26]. Because the control of gene expression in this parasite is mainly at the post-transcriptional level, further studies for the functional depiction of the PUF gene family in *T. cruzi* are still needed. Thus, we present here our efforts to study the PUF3 protein of *T. cruzi*, demonstrating that although it is not an essential protein, it influences mitochondrial transcripts, where changes in expression significantly affect mitochondrial morphology and function.

## 2 Materials and methods

### 2.1 Parasites

Wild-type (WT) *Trypanosoma cruzi* (M-RATTUS/CO/91/GAL-61.SUC; DTU TcI) parasites were maintained as epimastigotes at 28 °C in LIT medium supplemented with 10 % (vol/vol) heat-inactivated Fetal Bovine Serum (FBS) unless otherwise stated. Selection drugs were added to the medium at the following concentrations: 100 and 250 μg/mL G418 disulfate (Sigma Aldrich, St. Louis, USA) and 60 μg/mL Puromycin dihydrochloride from *Streptomyces alboniger* (Sigma Aldrich, St. Louis, USA).

### 2.2 Transfections

*Trypanosoma cruzi* epimastigotes were transfected by electroporation using the Amaxa® Cell Line Nucleofector® Kit T from Lonza with the X-001 program in the Amaxa nucleofector II b. For this, 5 × 10^7^ log-phase parasites were collected by centrifugation and resuspended in 100 μL of the kit solutions with the appropriate nucleic acid (plasmids, Cas9 enzyme, sgRNA, and DNA repair templates). After transfections, the parasites were allowed to recover for 24 h, and the drug selection was performed in 10 mL of the medium. Then, 100 μL of the parasite suspensions were aliquoted in 96-well plates and incubated at 28 °C. The transfected parasites were obtained approximately three weeks later.

### 2.3 *TcPuf3* knockout by CRISPR-Cas9

Double knockout lines were generated for the *TcPuf3* gene (C4B63_43g137) using the ribonucleoprotein (RNP) Cas9 from *Streptococcus pyogenes* (Synthego, California, USA). Two targeting CRISPR RNAs (crRNAs) were designed using the http://grna.ctegd.uga.edu/tool, synthesized by the Alt-R CRISPR service (Integrated DNA Technologies, USA), and used according to the manufacturer's instructions. Briefly, each crRNA was mixed with an equal concentration of tracerRNA to a final concentration of 50 μM. The mixture was then heated at 95 °C for 5 min and cooled at room temperature. Subsequently, 20 μM of the Cas9 enzyme was mixed simultaneously with both gRNAs and incubated for 20 min at room temperature to form the RNP complex. The homology repair template (HRT) was amplified from a plasmid containing the puromycin gene using two oligos, with either 30 nucleotides of the 5′-UTR or 3′-UTR from the *TcPuf3* plus 20 nucleotides of the Pyrimidine-rich sequence (Y) and puromycin, respectively. Three μg of the repair templates and 2 μL of the RNP complex were transfected using Amaxa-Nucleofector™ as described above. The selection was done with puromycin. As viability controls, parasites were transfected only with the Cas9 enzyme or with the RNP complex without HRT and were not subjected to selection with the drugs. Finally, allelic substitutions were confirmed by PCR amplification of the target gene using different combinations of primers. All the sequences are listed in the Supplementary Table S1.

### 2.4 Overexpression of *TcPuf3* in wild-type or knockout parasites

To overexpress the *Tc*PUF3 protein in wild-type or knockout parasites, the full-length gene (1686 bp) was amplified from wild-type clone DNA using specific primers bearing the human influenza virus hemagglutinin (HA) tag and ligated into the *Hind*III/*Xho*I site of the vectors pTEX or pTREX. Fifty μg of each plasmid was used to transfect epimastigotes from WT or knockout (addback) parasites, respectively, by electroporation as described above, and transformants were selected with 100 μg/mL of G418 and maintained with 250 μg/mL of the same drug. The *GFP*-HA gene cloned in the pTEX or pTREX vectors was used as a control. All the primers are listed in the Supplementary Table S1.

### 2.5 Immunolocalization of *Tc*PUF3 protein

Five hundred μL of epimastigotes in the exponential growth phase were incubated with MitoTracker™Red 1 mM (Invitrogen, Waltham, USA) at 28 °C for 30 min. Subsequently, parasites were fixed in 4 % formaldehyde, deposited in a 24-well plate on circular glass coverslips, and permeabilized with 0.3 % Triton X-100. A solution of filtered PBS plus BSA was used for blocking and labeling, and filtered PBS was used for washes. Anti-HA (1:1000, Proteintech, Rosemont, USA) was used as the primary antibody and anti-rabbit (1:100, Alexa 488, Waltham, USA) with Hoechst 333342 (1:750, Thermo Scientific, Waltham, USA) as the secondary antibody. Finally, the glass coverslip was removed from the plate and placed inverted on a slide with ten μL of FluorSave™ (Merck, Darmstadt, Germany). The slides were analyzed on the Fluoview™, 1000, OLYMPUS® confocal microscope, and the images were processed with the Fiji software (https://fiji.sc). Parasites overexpressing the *GFP*-HA gene in the pTEX vector were used as a control.

### 2.6 Evaluating the transcriptional regulation by PUF3 using RNA sequencing

RNA from 2 × 10^8^ epimastigotes was extracted using the *Quick*-RNA Miniprep Kit (Zymo Research, California, USA) and sent to the University of Oklahoma (Oklahoma, USA) for sequencing. For library preparation, mRNA was purified using poly-A tails and sequenced on the Illumina NovaSeq 6000 platform with paired reads methodology.

### 2.7 Bioinformatics analyses

Eighteen paired-end samples corresponded to three populations (WT, Δ*TcPuf3,* and pTEX*TcPuf3*) with three biological replicates. The quality of the reads obtained was evaluated using FastQC software. Trimmomatic software (v. 0.39) removed primers and adapters from the sequences and sequences with a Phred value lower than Q30. Reads were individually aligned against the *T. cruzi* Dm28c reference genome (2018) from DTU TcI using STAR software (v. 2.7.9a) with default parameters [27]. The.bam files obtained from the alignment were used by the featureCounts software with parameters '-T 20 -t protein_coding_gene -g gene_id -p -s 0 -a' to perform the transcript count per gene, based on the gff annotation file of the *T. cruzi* Dm28c reference genome (2018). Once the count's matrix was obtained, differential expression analysis was performed using the DESeq2 package and the WT as a control group for comparison. Genes with less than 32 counts were excluded, and genes were considered to be differentially regulated (DEG) when log2 was ≥0.58 (1.5-fold) and Benjamini-Hochberg FDR adjusted p-value of ≤0.05. Additionally, the data was transformed into transcripts per million (TPM) for transcript abundance analysis. This study predicted protein subcellular localization using DeepLoc-2.0, a state-of-the-art deep-learning model designed explicitly for this purpose [28]. To implement this methodology, protein sequences from our dataset were input into the DeepLoc-2.0 model, and the corresponding predictions and associated scores were extracted. Finally, Principal Component Analysis (PCA) was performed to ensure the quality of the count data. Positively and negatively regulated gene IDs obtained in DESeq2 were enriched using the TriTrypDB database (https://tritrypdb.org/tritrypdb/app). GO sequence distribution was analyzed for the three GO domains, biological processes (BP), molecular function (MF), and cellular component (CC), using the genome of *T. cruzi* Dm28c (2018). GO annotations were extracted and visualized as bubble plots using ggplot2 in R, disregarding the more general GO annotations. Additionally, functional annotation was performed using DAVID (Database for Annotation, Visualization, and Integrated Discovery, version 6.8).

### 2.8 PUF3 motif analysis

The genomic sequence of the 3′-UTR of each differentially expressed gene was downloaded from the TriTryp database (https://tritrypdb.org/tritrypdb/app). PUF3 motif analysis was performed using FIMO (https://meme-suite.org/meme/tools/fimol) [29]. For the analyses, we used a –thresh value of 0.05 to capture all motif configurations and filtered the output file to contain only the single motif with the highest FIMO motif score.

### 2.9 Western blots

Protein extracts from epimastigotes in the exponential growth phase were obtained with extraction buffer (Tris-HCl 20 mM pH 7.9, NaCl 100 mM, sucrose 0.25 M, EDTA 1 mM, EDTA 2 mM, Triton x-100 (v/v) 0.1 % and protease inhibitor (Roche, Mannheim, Germany)) by sonication. One hundred (100) μg of proteins were separated by vertical SDS-PAGE transferred to a nitrocellulose membrane. The gels were prepared with 2.5 mM 2,2,2-trichloro-1-ethoxyethanol to guarantee the uniform transfer of the proteins. The membranes were blocked with 3 % milk and incubated with the following primary antibody: anti-HA (1:1000-rabbit; Proteintech, Rosemont, USA), β-tubulin (1:1000-mouse; Santa Cruz Biotechnology, Dallas, USA), nitroreductase (TcNTRI-C4B63_56g60; 1:500-mouse), alcohol dehydrogenase putative (TcADH-C4B63_4g189; 1:250-rabbit), aldo-keto reductase (TcAKR-C4B63_175g10; 1:500-mouse), mitochondrial peroxiredoxin (TcMPX-C4B63_149g19, -C4B63_220g12; 1:500-rabbit) and prostaglandin F_2_alpha synthase (TcOYE-C4B63_2g187; 1:500-rabbit). The IRDye 800 CW Donkey anti-rabbit and IRDye 800 CW Goat anti-mouse IgG1 secondary antibodies (LI-COR Bioscience, Nebraska, USA) were used at 1:15,000 and blots were imaged with the Odyssey Classic Infrared System (Lincoln, USA) in both 700 and 800 nm channels. Results were normalized to β-tubulin in Image J software (https://imagej.net) [30], and relative quantification for each protein was obtained by comparing the intensity of normalized bands between the WT and the different phenotypes.

### 2.10 Northern blot

Fifteen μg of total RNA from epimastigotes were separated in a 1 % agarose/MOPS/formaldehyde gel. The RNA was then transferred to hybond-N + membrane (GE Healthcare, Munich, Germany) and hybridized with specific PCR probes (Table S1) previously labeled with “DIG DNA-labelling kit” (Roche, Basel, Switzerland). The hybridization was carried out as previously described by Ref. [31] using Church Gilbert buffer and the detection was done with the “DIG Luminescent Detection Kit” (Roche, Basel, Switzerland).

### 2.11 Proliferation curves

Two different methodologies were used. First, epimastigotes from WT, pTEX*GFP,* and pTEX*Puf3* phenotypes were seeded by triplicate at 1 × 10^6^ parasites/mL and were counted every 24 h for seven days in the Neubauer chamber. Second, 2.5 × 10^6^ epimastigotes/mL in the exponential growth phase from each *T. cruzi* phenotype were grown in triplicate in 96-well culture plates and maintained at 28 °C in the absence or presence of 30 μM of benznidazole (5× EC_50_). Cell proliferation was estimated for nine days from absorbance readings at 620 nm on the Multiskan Spectrum (Thermo Scientific, Waltham, USA). The values obtained were transformed to cell density using the linear regression equation previously determined with the same growth curve conditions. Finally, for all populations, the generation number and doubling time were calculated as described by Mejía-Jaramillo et al. (2009) [32]. To test the response to hydrogen peroxide (H_2_O_2_), 1 × 10^7^ epimastigotes/mL of WT, pTEX*GFP* and pTEX*Puf3* phenotypes were treated with 100 μM of this compound and counted at 72 h. The cell numbers were determined in a Neubauer chamber using the erythrosine vital stain to differentiate living and dead cells. The results were expressed as the percentage of growth compared to untreated cultures. The experiments were performed in triplicate.

### 2.12 Cellular differentiation from epimastigotes to metacyclic trypomastigotes

5 × 10^6^ epimastigotes/mL in the exponential growth phase were washed twice with 1× PBS, resuspended in TAU medium at a cell density of 5 × 10^7^ parasites/mL, and incubated for 2 h at 28 °C. Subsequently, parasites were resuspended in TAU 3AAG supplemented with 10 mM l-proline (Sigma Aldrich, Steinheim, Germany), 50 mM l-glutamate (Sigma Aldrich, Steinheim, Germany), 2 mM l-aspartate (Sigma Aldrich, Jiangsu, China), 10 mM d-glucose (Merck, Madrid, Spain) and incubated at 28 °C. The differentiation percentage was determined in the Neubauer chamber every 24 h for six days by the total count of parasites and metacyclic trypomastigotes. The experiments were performed in triplicate.

### 2.13 Infection assays

2 × 10^4^ VERO cells (ATCC-CCL-81) were cultured on glass coverslips in 24-well plates with RPMI medium supplemented with 2 % SFB at 37 °C and 5 % CO_2_. After 24 h, the cells were incubated in triplicate for 3 h with knockout metacyclic trypomastigotes from four days of differentiation. Forty-eight hours later, the cells were fixed with methanol and stained with Giemsa. Four hundred cells were selected to count the number of infected cells, and within this subset, the number of amastigotes per cell was determined. Counts were performed in triplicate using an OLYMPUS® optical microscope at 100× magnification.

### 2.14 Analysis of cellular respiration by oximetry

Respiratory characteristics of *T. cruzi* were determined by high-resolution respirometry using an Oxygraph-2k (Oroboros Instruments, Innsbruck, Austria). Epimastigotes (5 × 10^7^ parasites/mL) in the exponential growth phase were added to each chamber of the oxygraphy. Oxygen consumption rates were obtained using the O2k software DatLab 7.4 (Oroboros Instruments, Innsbruck, Austria). ATP-linked respiratory rate, FCCP (carbonyl cyanide 4- trifluoromethoxy phenylhydrazone) (Sigma-Aldrich, Jerusalem, Israel) -stimulated respiratory rate, H+ leak, non-mitochondrial respiration, and bioenergetic reserve were determined by successive addition of 4.5 μM (total) oligomycin A (Sigma-Aldrich, Jiangsu, China) as F_1_F_0_-ATPase inhibitor; 0.5 μM FCCP as mitochondrial uncoupler, and 4 μM antimycin A (Sigma-Aldrich, Jerusalem, Israel), from *Streptomyces* sp. as complex III inhibitor (Fig. S1). These concentrations were previously empirically established as optimal [33].

### 2.15 Transmission electron microscopy

Epimastigotes (1 × 10^6^ parasites/mL) between days 4–5 of growth were fixed with 2.5 % glutaraldehyde (Electron Microscopy Sciences, Pennsylvania, USA) for 1 h. Subsequently, the parasites were fixed with osmium tetroxide (Electron Microscopy Sciences, Pennsylvania, USA) for 20 min and dehydrated with 70 %, 95 %, and 100 % ethanol. Then, propylene oxide was added, and polymerization was performed in molds in an oven at 60 °C for 72 h. Finally, ultramicrotome cutting was done at 100 nm, and contrast was performed with uranyl acetate (Electron Microscopy Sciences, Pennsylvania, USA) and lead citrate. The samples were observed in a Tecnai F20 Super Twin TMP TEM (Eindhoven, Netherlands).

### 2.16 Flow cytometry analysis

Epimastigotes (1 × 10^6^ parasites/mL) in the exponential growth phase were used for cell cycle analysis, mitochondrial membrane potential (ΔΨm), and reactive oxygen species (ROS) determination as described previously [34]. For the cell division, the parasites were synchronized with 10 mM Hydroxyurea (Sigma Aldrich, St. Louis, USA) for 16 h, labeled with a propidium iodide solution (PI; Invitrogen, Waltham, USA) and treated with 10 μg/mL RNAase. To determine membrane potential, labeling was done with 0.4 μg/mL 3,3 - dihexyloxacarbocyanine iodide (DiOC_6_(3); Invitrogen, Waltham, USA) and 20 μg/mL PI and unlabeled *T. cruzi* parasites were used as compensation controls. DiOC_6_-positive and PI-negative cells were considered alive, DiOC_6_-negative and PI-negative cells with mitochondrial potential damage, and DiOC_6_-positive and PI-positive cells with membrane damage. Finally, to determine ROS, parasites were labeled with 0.1 μM of 2,7-Dichlorofluoroscin Diacetate (H_2_DCFDA; Invitrogen, Waltham, United States); as a positive control, parasites were treated with 100 μM of H_2_O_2_, for 20 min. The mean fluorescence intensity was measured in the positive DCF-DA parasite population.

In all the experiments, readings were performed on a BD LSRFortessa Cell Analyzer flow cytometer (BD Headquarters, New York, USA), and data was analyzed using FlowJo 10.8 software. Ten thousand events were acquired in the region previously established as the parasite population, and all experiments were performed in triplicate.

### 2.17 Statistical analysis

The data were visualized and analyzed in GraphPad Prism 8.0. One-way ANOVA with multiple comparisons was used for metacyclogenesis, flow cytometry, transmission electron microscopy, oxygen consumption associated with ATP production, and bioenergetic reserve. A two-way ANOVA was performed to determine the proliferation and the significance of bioenergetic reserve ATP-linked respiration rate. The Tukey correction was employed for the oximetry experiments, and the Sidak *p-value* correction for the other experiments. Values are presented as mean ± SD. Statistical significance was determined when p < 0.05.

## 3 Results

### 3.1 The *TcPuf3* gene is not essential in *Trypanosoma cruzi*

To determine whether PUF3 was essential in *T. cruzi*, we used the *Sp*Cas9 ribonucleoprotein to delete the gene's coding sequence. Three clones were obtained in the 96 well plates selected with puromycin after transfections with the RNP complex and HRTs. The DNA was extracted, and different PCRs were carried out. The result for all clones confirmed the absence of *Puf3* amplification and the presence of the puromycin gene at the corresponding locus. Thus, we confirmed that the parasites obtained were double knockout for *Puf3* (Fig. S2), which indicates that this gene is not essential in *T. cruzi* epimastigotes. Then, we obtained amastigotes and trypomastigotes from one clone, confirming the non-essentiality of this gene in any *T. cruzi* life-cycle stage (Fig. S3). Finally, we selected this clone to study the role of PUF3 in the parasite.

### 3.2 The *Tc*PUF3 is localized in the cytoplasm in *Trypanosoma cruzi*

To determine whether PUF3 was in the nucleus or cytoplasm, we obtained parasites expressing C-terminally HA-tagged PUF3 confirmed by Western blot (Fig. S4A). Using immunofluorescence microscopy, we observed that PUF3-HA was spread throughout the cytosol with no detectable signal from the nucleus, with the comparable signal as GFP-HA tagged protein (Fig. S4B). We also confirmed the level of expression of PUF3 by Western blot was not that high compared with the expression of C-terminally HA-tagged GFP (Fig. S4A), although we detected the overexpression of the mRNA by northern blot (Fig. S4C). This may suggest that the correct level of PUF3 is required for optimal growth and that an excess might be toxic, as was shown in the related parasite, *T. brucei* [24] and in *S. cerevisiae* [21].

### 3.3 *Tc*PUF3 changes the level of mRNAs of mitochondrial genes encoded in the nucleus

Since PUF proteins are involved in expression regulation when bound to mRNAs, we investigated if the depletion or the ectopic expression of PUF3 affected mRNA levels. Principal Component Analysis (PCA) was employed to explore sample variability within the RNA-Seq dataset (Fig. 1A). The PCA reveals the grouping of biological replicates based on their respective phenotypes. Furthermore, a notable observation is the greater separation of parasites with a *TcPuf3* gene knockout compared to wild-type parasites. This observation implies that the gene expression patterns for the knockout parasites are the most dissimilar among the analyzed groups. The differentially expressed genes analysis showed that the knockout and the overexpressor impact the expression of a comparable number of genes (238 and 187, respectively) (Fig. 1B; Table S2). However, the crucial distinction lies in the direction of the change in expression. In the knockout, a more significant proportion of genes was negatively regulated (166 out of 238). In contrast, in the overexpressor, positively regulated genes were predominant (149 out of 170) (Fig. 1B). In addition, an overlap of 11 genes was observed between the two mutants (Table S2).

**Fig. 1:**
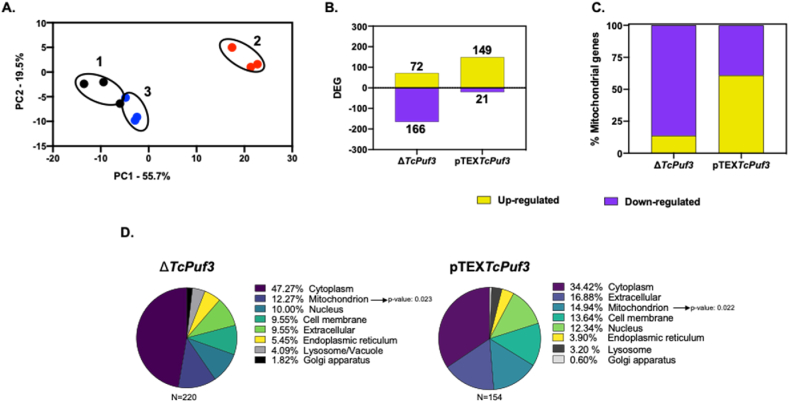
Impact of *TcPuf3* knockout and overexpression on gene expression patterns and subcellular localization. A. PCA analysis of the RNA sequencing data from three biological replicates of 1, WT; 2, Δ*TcPuf3*; 3, pTEX*TcPuf3*. **B.** Analysis of differentially regulated genes (DEG) from Δ*TcPuf3* and pTEX*TcPuf3* compared with the WT parasites. **C.** Percentage of up-regulated and down-regulated mitochondrial genes found in the Δ*TcPuf3* and pTEX*TcPuf3* genotypes. **D.** Analysis of the enrichment of protein subcellular localization of the DEG from Δ*TcPuf3* and pTEX*TcPuf3*.

When we predicted the subcellular location of the differentially expressed genes, the results revealed a notable representation of mitochondrial genes, with 27 for the Δ*TcPuf3* and 23 for pTEX*TcPuf3* (Fig. 1C and D). This finding indicates that *Tc*PUF3 regulates the expression of targeted genes associated with mitochondria, highlighting a potential role in energy production, cellular metabolism, or other mitochondrial-related functions.

### 3.4 Mitochondrial targets exhibit an enrichment of the PUF3 binding domain

To ascertain the direct targets of PUF3 among the identified genes, we searched for the presence of the PUF3 UGUAYAUW binding motif in the 3′-UTR (depicted in Fig. 2A). Our analysis revealed that, of the 238 differentially expressed genes in the knockout parasites, 25 % possess at least one binding site for PUF3 in their 3′-UTR. Interestingly, a parallel proportion of 18.82 % was observed in the overexpressor (Fig. 2B). Subsequently, we investigated if the subset of genes with a PUF3 binding site was associated with mitochondrial functions. For the knockout condition, approximately 33 % of genes with a PUF3 binding site exhibit mitochondrial localization, whereas for the overexpression condition, we found 17.39 % (Fig. 2C). For further insight into the identity of these genes, a scatterplot integrating the degree of regulation (fold change; FC) and abundance (Average of Normalized Counts) for all genes regulated by PUF3 under knockout and overexpression conditions was generated (Fig. 2D). In Δ*TcPuf3* parasites, most of the mitochondrial genes with putative PUF3 binding sites were found to encode proteins that have not yet been characterized. Notably, the knockout increases the expression of two genes associated with the electron transport chain (ATPase and ATP synthase alpha subunit) and a gene involved in cellular metabolism (Thiamin pyrophosphokinase 1). In the case of pTEX*TcPuf3*, we found four genes with putative PUF3 binding sites, of which two were hypothetical proteins, and two were related to mitochondrial structure, such as transmembrane protein 65 (Fig. 2D). Noteworthy regulation includes tryparedoxin peroxidases, recognized for its role as a mitochondrial antioxidant [35]. However, no binding sites for PUF3 were identified in these genes. Our findings highlight that modulation of PUF3 expression regulates many non-overlapping genes. This implies that the effects of PUF3 modulation on gene regulation depend on its intracellular concentration. Furthermore, our results indicate that the repertoire of regulated genes encompasses those with a binding site for PUF3 and those without, suggesting the existence of indirect targets.

**Fig. 2:**
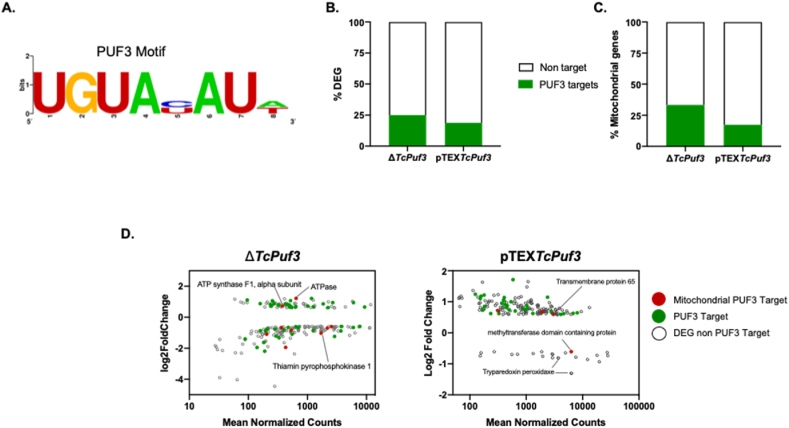
Target genes of PUF3 in knockout and overexpressor parasites. A. PUF3 binding motif in the mRNAs 3′-UTR. **B and C.** Analysis of target binding motifs in the differentially regulated genes (DEG) (B) or mitochondrial genes (C) from Δ*TcPuf3* and pTEX*TcPuf3*. A threshold value 0.05 was used to capture all motif configurations, and the output file was filtered to contain only the single motif with the highest FIMO motif score. **D.** Correlation between the fold change and the mean of Normalized Counts for all genes regulated by Δ*TcPuf3* (left panel) and pTEX*TcPuf3* (right panel) genotypes.

### 3.5 Gene ontology enrichment in Δ*TcPuf3* and pTEX*TcPuf3* cells supports the role of PUF3 in mitochondrial function

The gene ontology (GO) enrichment analysis performed on genes differentially expressed in Δ*TcPuf3* cells showed 160 GO terms distributed in three domains, 95 belonging to biological processes, 53 to molecular function, and 12 to cellular components (Table S3). Fig. S5 shows the 30 GO terms with the highest fold enrichment for each domain; this value was obtained from the total number of genes differentially expressed in the knockout parasites compared with the total gene background of the reference strain *T. cruzi* Dm28c belonging to the same GO term.

Gene ontology analysis revealed that Δ*TcPuf3* phenotype impacts the expression of genes related to bioenergetic processes, especially those linked to acetate metabolism, cofactors such as the heme group, and coenzymes such as thiamine. Additionally, ten GO terms related to biological processes involved in DNA repair were found, modulated by SMC5-SMC6 complex localization factor protein 1 (C4B63_16g152), which plays a vital role in the repair of DNA double-strand breaks through homologous recombination of sister chromatids.

In terms of molecular functions, routes involved in transmembrane transport based on inorganic ion antiporter systems, routes related to purine nucleoside phosphorylation, thiamine phosphorylation, and activation, and AMP precursor adenylosuccinate synthesis were also observed. Terms were associated with AMP binding, especially with activity leading to acetyl-CoA synthesis.

The GO enrichment analysis performed on pTEX*TcPuf3* cells showed 53 GO terms distributed in three domains: 33 belonging to molecular function, 15 to biological processes, and five to cellular components (Table S4). As shown in Fig. S6, pTEX*TcPuf3* cells showed an impact on terms related to ubiquitination processes (C4B63_31g76), tRNA modification (C4B63_31g48), and signal peptide processing (C4B63_31g105), enriched by overexpressed genes involved in fundamental protein synthesis processes. Additionally, terms associated with flagellum assembly and organization (C4B63_31g51 and C4B63_31g52) were enriched. Regarding molecular function, over-regulated genes were related to the maturation and generation of functional mRNA (C4B63_59g125). The terms associated with the under-regulated genes involved antioxidant activity and protection against oxidative stress (C4B63_19g43, C4B63_60g172, C4B63_26g253, C4B63_26g257).

### 3.6 *Tc*PUF3 affects mitochondrial morphology and cellular respiration

Considering that the transcriptomic results suggest an essential representation of mitochondrial genes, we wanted to evaluate whether overexpression or knockout of *TcPuf3* would lead to changes in both mitochondrial structure and cellular respiration. When mitochondria from WT (Fig. 3A and B), Δ*TcPuf3* (Fig. 3C and D) and pTEX*TcPuf3* (Fig. 3E and F) parasites were analyzed by transmission electron microscopy (TEM), it was observed that the overexpressor had an abnormal mitochondrial morphology, with the appearance of a considerable gap between the membrane and the kinetoplast (Fig. 3A–F, Supplementary File S1). This abnormal morphology was visible as a notable swelling, with decreased electron density in the matrix and a significantly increased mitochondrial membrane width (p < 0.0001) with a 3-fold increased size compared to WT and knockout parasites (Fig. 3G).

**Fig. 3:**
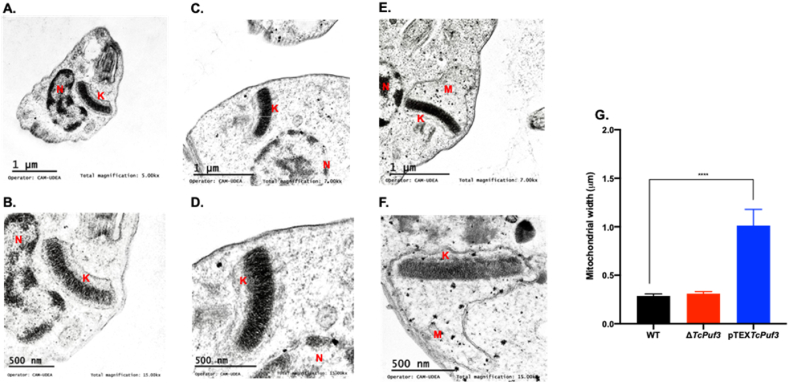
Transmission electron microscopy analysis of *Trypanosoma cruzi* epimastigotes from WT (A and B), Δ*TcPuf3* (C and D), and pTEX*TcPuf3* (E and F) populations. The WT presents typical morphology of the mitochondria (M), nucleus (N), and kinetoplast (K). Scale bar = 500 nm and one μm. **G.** Mitochondrial width in μm of each *T. cruzi* population analyzed in 12 cells per phenotype. Statistical significance was determined by one-way ANOVA and performed in GraphPad Prism 8.0. ****p < 0.0001.

In addition to the morphological changes observed in the mitochondrion, the functionality of this organelle was assessed by studying cellular respiration. The Δ*TcPuf3*, addback, and pTEX*TcPuf3* had lower efficiency in routine respiration and the electron transport system capacity. The absence or overexpression of PUF3 decreased routine respiration by 20 % and 40 %, respectively. The inhibition of mitochondrial F_1_F_0_-ATPase by oligomycin A resulted, on average, in a 53 % decrease of the O_2_ consumption rate, without significant difference between populations. On the other hand, the uncoupler FCCP increased the oligomycin A-sensitive O_2_ consumption rate by a factor of 3.5 for Δ*TcPuf3,* WT, and pTEX*GFP*; 3 for pTEX*TcPuf3* and 2.4 for the addback (Fig. 4A)*.* Surprisingly, a statistically significant difference was observed in oligomycin A, FCCP-stimulated and routine O_2_ consumption rates in pTEX*TcPuf3* and addback, suggesting the lack of ATP-linked respiratory rate and bioenergetic reserve capacity in the exponential phase of these parasites (Fig. 4B and C). Fig. 4D shows the relationship between ATP-linked respiratory rate and bioenergetic reserve capacity for the genotypes. In addition, inhibition of mitochondrial complex III by antimycin A was observed to decrease routine and FCCP-stimulated O_2_ consumption rates, on average, by 94 % for Δ*TcPuf3*, 87 % for the addback and 89 % for pTEX*TcPuf3*. Therefore, 6 % in Δ*TcPuf3*, 13 % in the addback and 11 % in pTEX*TcPuf3* of the routine O_2_ consumption rate appear to be related to non-mitochondrial processes.

**Fig. 4:**
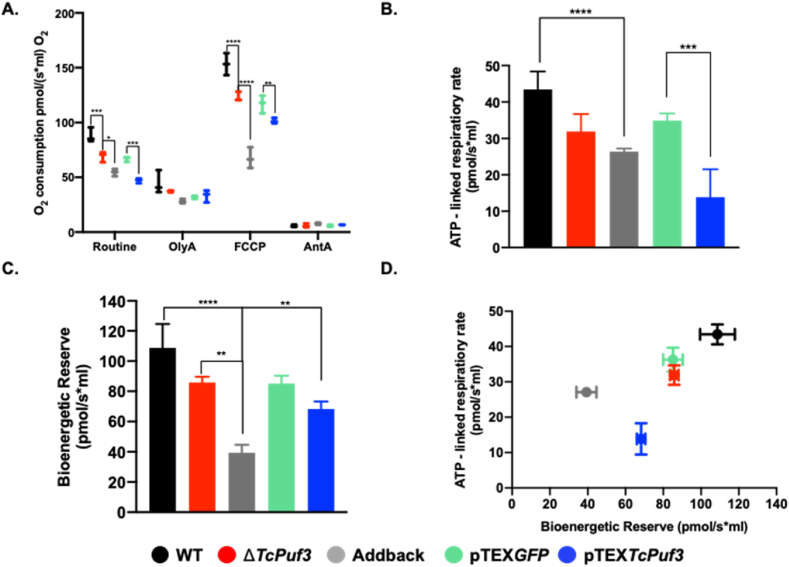
Measurement of bioenergetic parameters in *ΔTcPuf3* and pTEX*TcPuf3* populations. A. Oxygen consumption rates were obtained using the O2k software DatLab 7.4. OlyA: oligomycin A; FCCP: carbonyl cyanide 4- trifluoromethoxy phenylhydrazone; AntA: antimycin A. **B.** Oxygen consumption associated with ATP production **C.** Bioenergetic reserve. **D.** Scheme of the relationship between the parameters associated with ATP production and bioenergetic reserve. Statistical significance was determined in GraphPad Prism 8.0 by comparing the Δ*TcPuf3*, addback, and pTEX*TcPuf3* with the respective control population (WT or pTEX*GFP*) using the two-way ANOVA (A) and one-way ANOVA (C and B) with a *p-value* correction using a multiple comparison Tukey method. ****p < 0.0001; ***p < 0.001; **p < 0.01. Wild-type parasites (WT) are represented in black; Δ*TcPuf3* in red; addback in gray; pTEX*GFP* in green, and pTEX*TcPuf3* in blue.

### 3.7 Changes in *Tc*PUF3 expression affect mitochondrial membrane potential and ROS production

Considering that the previous results showed drastic changes in different mitochondrial parameters, we wanted to explore if there were other alterations related to this organelle in Δ*TcPuf3* and pTEX*TcPuf3*. When the mitochondrial membrane potential (ΔΨm) was measured by flow cytometry with DiOC_6_, we found a slight increase in the parasites overexpressing *Puf3* (Fig. 5A and B).

**Fig. 5:**
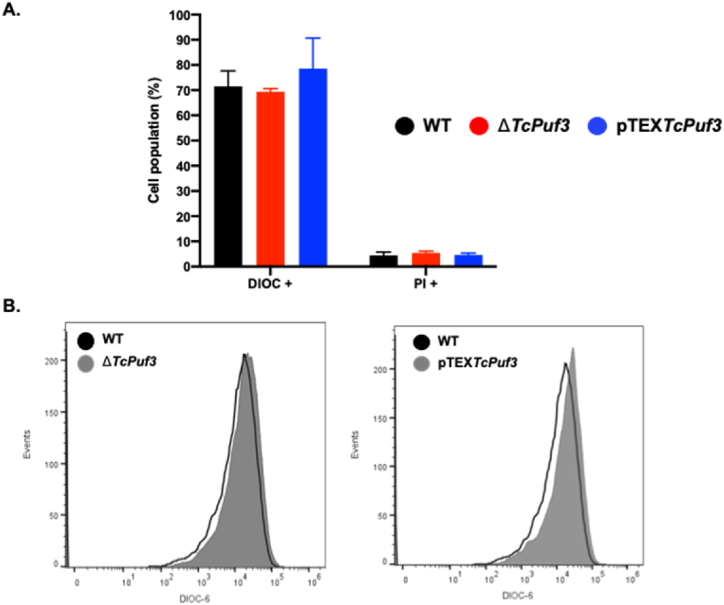
Measurement of mitochondrial membrane potential. **A and B.** Flow cytometry analysis and histograms, respectively, show changes in mitochondrial membrane potential (ΔΨm) measured by DiOC_6_ staining in different genotypes. Statistical significance was determined by one-way ANOVA and performed in GraphPad Prism 8.0.

In addition, the ROS levels, measured by the percentage of DCFDA-positive cells by flow cytometry, differed in these populations. Thus, in the Δ*TcPuf3* and pTEX*TcPuf3* populations, a statistically significant increase in total ROS production was observed at 39.5 % and 38 %, respectively, compared to 4.5 % in the WT population (Fig. 6A and B), supporting the substantial reduction in respiratory rates and mitochondria swelling observed in these parasites. To investigate further the ROS production in these populations, they were stimulated with 100 μM H_2_O_2_. Surprisingly, while the WT population increased ROS production by 8×, the PUF3 phenotypes only increased by approximately 1.5×, indicating that these parasites intrinsically have more ROS (Fig. 6A). Finally, we calculate the survival percentage of WT, pTEX*GFP* and pTEX*TcPuf3* after 72 h exposure with 100 μM H_2_O_2_ and notice significant differences in the survival of the *Puf3* overexpressing parasites compared with the controls (Fig. 6C).

**Fig. 6:**
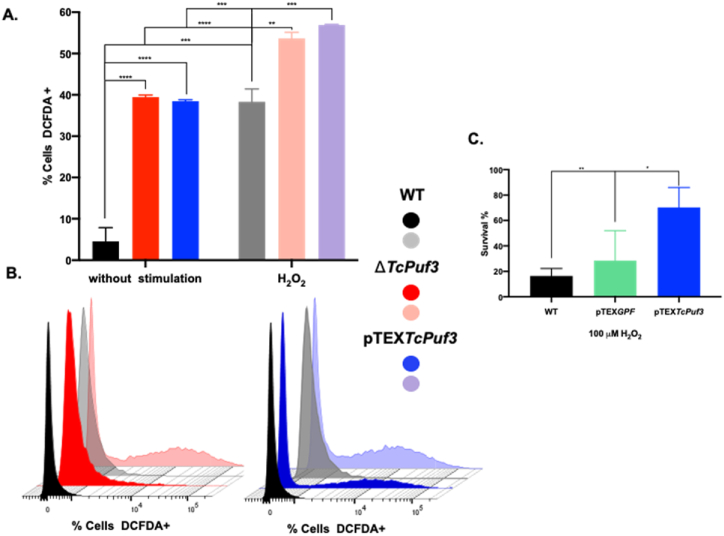
Measurement of ROS production. **A and B.** Flow cytometry analysis and histograms, respectively, of ROS assessed by H_2_DCFDA labeling. The parasites were untreated or stimulated with hydrogen peroxide (H_2_O_2_) as a ROS inducer. Wild-type parasites (WT) are represented in black and gray (exposed to H_2_O_2_); Δ*TcPuf3* in red and pink (exposed to H_2_O_2_), and pTEX*TcPuf3* in blue and purple (exposed to H_2_O_2_). **C.** Survival percentage of epimastigotes after the exposure with H_2_O_2_ after 72 h. Statistical significance was determined by one-way ANOVA and performed in GraphPad Prism 8.0. ****p < 0.0001; ***p < 0.001; **p < 0.01; *p < 0.05.

### 3.8 Changes in *Tc*PUF3 expression alters mitochondrial protein expression in *Trypanosoma cruzi*

Next, we tested the expression of different mitochondrial proteins using Western blot. In both knockout and overexpressed parasites, the levels of some proteins changed their expression compared to the control. Thus, in the knockout parasites, the following proteins were found down-expressed: *Tc*ADH (1.4×), *Tc*OYE (1.4×), *Tc*AKR (1.4×), and *Tc*MPX (1.5×). The proteins *Tc*OYE and *Tc*AKR were overexpressed by 1.3× in the parasites with ectopic expression of PUF3, while *Tc*NTRI (2×), *Tc*ADH (1.4×) and *Tc*MPX (2.1×) were downregulated (Fig. S7).

### 3.9 Changes in *Tc*PUF3 expression negatively affect *Trypanosoma cruzi* growth and response to Bz

To obtain further information about the role of PUF3 in *T. cruzi*, we examined whether there were any biological alterations in these phenotypes compared with the WT. The growth of Δ*TcPuf3*, addback, and pTEX*TcPuf3* epimastigotes revealed statistically significant differences in proliferation compared with the control (WT or pTEX*GFP*). The results showed that both the depletion and overexpression of *Puf3* in *T. cruzi* affected not only the number of parasites but also the doubling time between days 2 and 3 (Fig. 7A and B), where values of 34 h were obtained for Δ*TcPuf3,* 24h for pTEX*TcPuf3*, 20 h for WT and 45h for the addback. This result was confirmed by cell cycle analysis using flow cytometry, where after 16 h of HU treatment, the WT parasites reached almost 80 % of the cells in G1. At the same time, the Δ*TcPuf3* and pTEX*TcPuf3* genotypes were only 57 % and 65 %, respectively. The remaining parasites were in other cell cycle phases (Fig. S8).

**Fig. 7:**
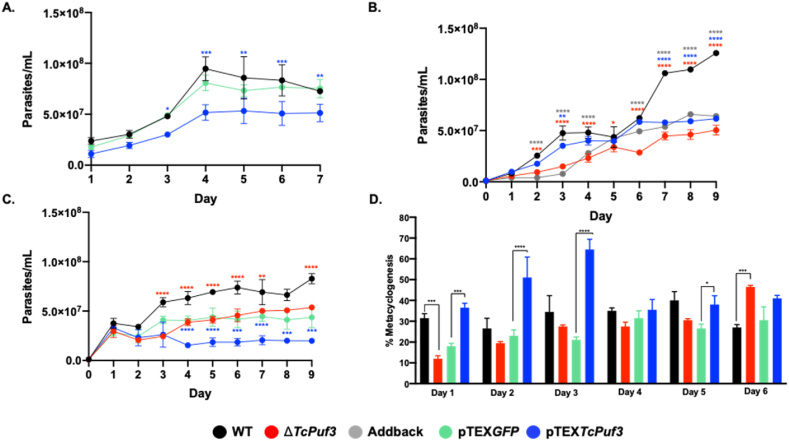
Biological characterization of *TcPuf3* phenotypes. A. WT, pTEX*GFP,* and pTEX*TcPuf3* epimastigotes proliferation curves assessed by counting in Neubauer chamber every 24 h for seven days. No differences were found between WT and pTEX*GFP*. **B**. WT, Δ*TcPuf3*, addback and pTEX*TcPuf3* epimastigotes proliferation curves. Parasite growth was determined relative to the control population. **C**. Effect of benznidazole on the proliferation of Δ*TcPuf3*, addback and pTEX*TcPuf3* epimastigotes. Parasite growth was compared to the WT and pTEX*GFP*, exposed to 30 μM of Bz. **D.***In vitro* metacyclogenesis for Δ*TcPuf3* and pTEX*TcPuf3* populations. The parasites were maintained in the TAU 3AAG differentiation medium and counted every 24 h. Statistical significance was determined using two-way ANOVA (A, B, and C) and one-way ANOVA (D) with Sidak multiple comparison correction, performed in GraphPad Prism 8.0. ****p < 0.0001; ***p < 0.001; **p < 0.01; *p < 0.05. Wild-type parasites (WT) are represented in black; Δ*TcPuf3* in red; addback in gray; pTEX*GFP* in green, and pTEX*TcPuf3* in blue.

Since we found an essential representation of differentially expressed transcripts that localize in the mitochondria, we also decided to evaluate the response to benznidazole. This drug is activated by mitochondrial reductases. The drug exposure resulted in highly severe growth retardation. Interestingly, pTEX*TcPuf3* parasites were the ones that presented the highest sensitivity to the drug and the most significant differences with the controls (Fig. 7C).

### 3.10 *Tc*PUF3 affects *in vitro* metacyclogenesis in *Trypanosoma cruzi*

*T. cruzi* metacyclogenesis is generally accompanied by alterations in protein expression. Therefore, we tested whether parasites with changes in PUF3 expression (Δ*TcPuf3* and pTEX*TcPuf3*) altered the differentiation of epimastigotes to trypomastigotes. In the Δ*TcPuf3* parasites, the differentiation process was decreased concerning the control from day 1–5 and only reached a maximum of 40 % of metacyclogenesis on day 6. In contrast, in pTEX*TcPuf3* overexpressing parasites had a higher differentiation from day 1 with a percentage of metacyclogenesis of 40 % and a maximum peak of differentiation on day 3 with a rate above 60 %, which was not reached by any of the phenotypes evaluated (Fig. 7D).

## 4 Discussion

The PUF proteins regulate gene expression post-transcriptionally in most eukaryotic cells. In this paper, we studied the role of PUF3 protein in the parasite *Trypanosoma cruzi*. We compared the transcriptional profile and some biological features from knockout parasites and those overexpressing the *Puf3* gene. Our data suggests a role of PUF3 in mitochondrial structure, electron transport chain assembly, and function of this organelle. Firstly, a significant number of nuclear genes encoding for mitochondrial proteins are strongly regulated by PUF3, as was demonstrated by RNA-seq. Secondly, the ultrastructural changes in mitochondria shape were observed by electron microscopy; thirdly, the bioenergetic parameters measured in the presence of several inhibitors support the disassembly of the electron transport chain; and fourth, the effect on ROS production and response to H_2_O_2_. Additionally, several biological features were altered, such as growth, metacyclogenesis, and response to benznidazole, all of which depend on mitochondrial energetic metabolism.

The evidence about the direct role of PUF3 in mitochondrial mRNA targeting in yeast includes, among others, the coimmunoprecipitation of mitochondria-localized mRNAs with PUF3 [36,37] and that specific PUF3-binding sites in the 3′UTRs of these mRNAs are essential for the mitochondrial localization of the mRNA [36]. Some other experiments have shown that PUF3 overexpression in yeast leads to impaired respiratory growth [36], but so does the deletion of PUF3 [11,37]. Although a puf3D single mutant is only modestly compromised for growth on a non-fermentable carbon source such as glycerol [37], PUF3 becomes essential for respiratory growth on glycerol in a yeast strain lacking the mitochondrial import receptor Tom20 [11].

According to the RNA-seq data, in our study, 27 out of 238 genes regulated corresponded to mitochondrial proteins in parasites lacking the PUF3 protein, reinforcing its role in controlling mitochondrial activity. Similar results have been found in *S. cerevisiae,* where this protein represses the expression preferentially of mRNAs of nuclear-encoded mitochondrial proteins [3,7,8,38]. Also, it has been observed that this protein controls the early steps of mitochondria biogenesis in yeast, where cytochrome c-oxidase assembly requires around 18 proteins, of which 17 are targets of PUF3 [36].

Conversely, it is relevant to highlight that the PUF3 protein does not exclusively regulate nuclear genes encoding mitochondrial proteins. In *S. cerevisiae,* PUF3 may also regulate proteins functioning in the nucleus and nucleolus [39]. Analogously, we found that both *T. cruzi* phenotypes Δ*TcPuf3* and pTEX*TcPuf3* regulate mRNA mitochondrial encoding proteins, nuclear, cytosolic, and proteins from other organelles. In this sense, our results open new challenges in understanding gene regulation in trypanosomatids.

For all PUFs, whole-transcriptome analysis has revealed many putative partner transcripts. However, only a moderate number of mRNAs have been validated as targets by direct assays. There are some reports for PUF3 from yeast where most of the putative mRNA partners are relevant targets *in vivo* [36,37], but in other cases, this does not occur [2,40,41]. For example, only two out of 20 putative targets of PUFs in yeast showed differences in half-lives in *Puf* mutants [41] and seven out of 15 in another study [40]. In *T. cruzi*, the overexpression of *Tc*PUF6 decreases the mRNA levels of its targets [25,26]. The same has been found in the case of *T. brucei,* where both the over or reduced expression of different *Tb*PUF proteins impact the levels of target mRNA [10]. Here, we complemented our transcriptome analyses by searching for the UGUAYAUW binding motif and found, for more than 20 % of the regulated genes, the presence of such a motif in the 3′UTRs. Thus, although it seems very probable that *Tc*PUF3 directly or indirectly controls the expression of these mRNA, future experiments will need to validate the specific mRNA targets and test how PUFs regulate their expression and localization in *Trypanosoma cruzi*.

Furthermore, the general analysis of regulated genes in Δ*TcPuf3* and pTEX*TcPuf3* parasites suggests that there is no correlation between the increase or decrease in gene abundance. This could indicate that other factors, such as other PUF proteins or several RNA-binding proteins, also regulate the expression of these genes. Future studies must be performed to decipher the role of these proteins in the genetic regulation of this parasite.

We also found that while the *T. cruzi* PUF3 protein is not essential, its absence does cause alterations in the cell division and the differentiation process to trypomastigotes. In *T. brucei*, the reduced PUF3 expression caused a significant loss of fitness associated mainly with early cell differentiation, although depletion had no significant effect on the transcriptome [24]. In contrast, in our study, many transcripts were regulated without the PUF3 protein, which could be responsible for such changes. An interesting downregulated transcript found in this genotype was related to the repair of DNA double-strand breaks (C4B63_16g152). This result could explain the delay in progression through the cell cycle of these parasites, as the lack of this type of protein may cause the parasites to take longer to repair DNA to continue the cell division.

Additionally, we observed that ectopic expression of this protein in *T. cruzi* can more drastically alter the mitochondrial structure and cell differentiation. Similar results were obtained in *T. brucei*, where the overexpression of this protein had deleterious effects in procyclic forms [24]. This aspect is interesting since increased expression of PUF3 can cause excessive repression of key mRNAs in cell division and differentiation. However, these aspects must be studied in future studies to demonstrate the fundamental role of PUF3 in these processes.

Another aspect to highlight is the response to oxidative stress. *Trypanosoma cruzi Puf*3 mutants respond better to hydrogen peroxide than WT. This result is not surprising considering that these parasites already produce more ROS, which may prepare them to respond better to exposure to this compound. Similarly, *S. cerevisiae* with a deletion of this gene presents more robust growth under oxidative stress conditions [42]. Moreover, when these parasites are exposed to H_2_O_2_, they do not significantly increase ROS production, probably due to the downregulation of two tryparedoxin peroxidases (C4B63_26g257 and C4B63_26g253) found by RNA-seq.

In addition, when we exposed both Δ*TcPuf3* and pTEX*TcPuf3* to different concentrations of Bz, we observed an adverse effect that was much more significant on the overexpressor. Bz metabolism in *T. cruzi* has been associated with mitochondrial proteins, including *Tc*NTRI, *Tc*ADH, *Tc*AKR, and *Tc*OYE, among others [34,43,44]. Interestingly, these proteins were differentially expressed in the parasites overexpressing PUF3. Given that we generally found an altered mitochondrial function and shape in our PUF3 mutants, it is not surprising that these genotypes were more susceptibility to Bz.

The other aspect to consider is the mitochondria structure. PUF3 overexpressor *T. cruzi* parasites exhibited severe ultrastructural changes, such as mitochondrial swelling. Scientific evidence reveals that different types of compounds can cause these changes and alterations in mitochondrial ultrastructure in trypanosomatids, such as ketoconazole, ruthenium, acriflavine, antricide, pentamidine, puromycin, suramin and mapharside [[45], [46], [47], [48]], showing that these parasites are under much stress which explains all the genetic and biological changes. Also, these changes have been associated with the interference of mitochondrial activity, shown by changes in the ΔΨm [45]. However, the absence of ultrastructural evidence in the knockout parasites does not exclude effects on other aspects of physiology and metabolism that may affect other organelles. Therefore, the mitochondrial membrane potential changes observed in *T. cruzi* parasites overexpressing PUF3 could result from inhibition of the electron transport chain (decrease) or permeabilization of the inner membrane (decrease). In the same way, de Macedo-Silva et al. (2013) demonstrated that *L.* (L.) *amazonensis* promastigotes presented a collapse of the mitochondrial membrane potential associated with intense mitochondrial swelling, disorganization, and rupture of mitochondrial membranes [49].

It is essential to highlight that although the results obtained from bioinformatics analyses and the biological and functional characterization of knockout and overexpressor parasites may appear to be unrelated and inconsistent, in terms of messenger RNA regulation, the absence and excess of PUF3 are not necessarily related. Thus, while PUF3 absence can be partially substituted by similar proteins, corroborated by the non-essentiality of this protein in *T. cruzi*, excess can lead to an over-regulation of transcripts, even non-specific ones, which in the long run could be toxic for the organisms or have more noticeable effects, as in our case of the mitochondria swelling obtained in the pTEX*Puf3*. As already mentioned, different responses are observed when PUF3 is deleted or overexpressed in yeast [21,39].

Finally, recently, Asencio et al. (2024), demonstrated that transfection of a ribonucleoprotein complex, composed of recombinant *sp*Cas9 and *in vitro* synthesized sgRNA, produces rapid and efficient genetic modifications for single genes of *T. brucei*, *T. congolense*, and *Leishmania* spp [50]. Here, we showed that this approach also functions in *T. cruzi*, opening new editing possibilities in this parasite.

Overall, our data suggest a crucial role for the PUF3 protein in maintaining the structure and function of *T. cruzi* mitochondria. Likewise, we propose PUF3 as a strategic protein regulating genes destined for other organelles and in fundamental cell processes such as cell respiration, response to oxidative stress, and defense against drugs.

## Additional information

No additional information is available for this paper.

## Data availability statement

Transcriptomic data are available at https://dataview.ncbi.nlm.nih.gov/object/PRJNA1089800?reviewer=dgbaff4go6ndb08s71jt89iert.

## CRediT authorship contribution statement

**Ana María Mejía-Jaramillo:** Writing – review & editing, Writing – original draft, Visualization, Validation, Supervision, Methodology, Investigation, Funding acquisition, Formal analysis, Data curation, Conceptualization. **Geysson Javier Fernandez:** Writing – original draft, Formal analysis. **Hader Ospina-Zapata:** Investigation, Formal analysis. **Ana Milena Murillo:** Investigation. **Dianny Elizabeth Jimenez:** Investigation. **Luis A. Gómez:** Resources, Investigation, Formal analysis. **Omar Triana-Chávez:** Writing – review & editing, Writing – original draft, Visualization, Validation, Supervision, Resources, Project administration, Investigation, Funding acquisition, Formal analysis, Data curation, Conceptualization.

## Declaration of competing interest

The authors declare the following financial interests/personal relationships which may be considered as potential competing interests: Ana María Mejía-Jaramillo and Omar Triana-Chávez report financial support was provided by 10.13039/501100005278Universidad de Antioquia and 10.13039/501100013409SGR Grant BPIN 2020000100479, Colombia. Hader Ospina-Zapata reports financial support was provided by Grant 1115-918-91933 Minciencias-Colombia.

## Figures and Tables

**figs1:**
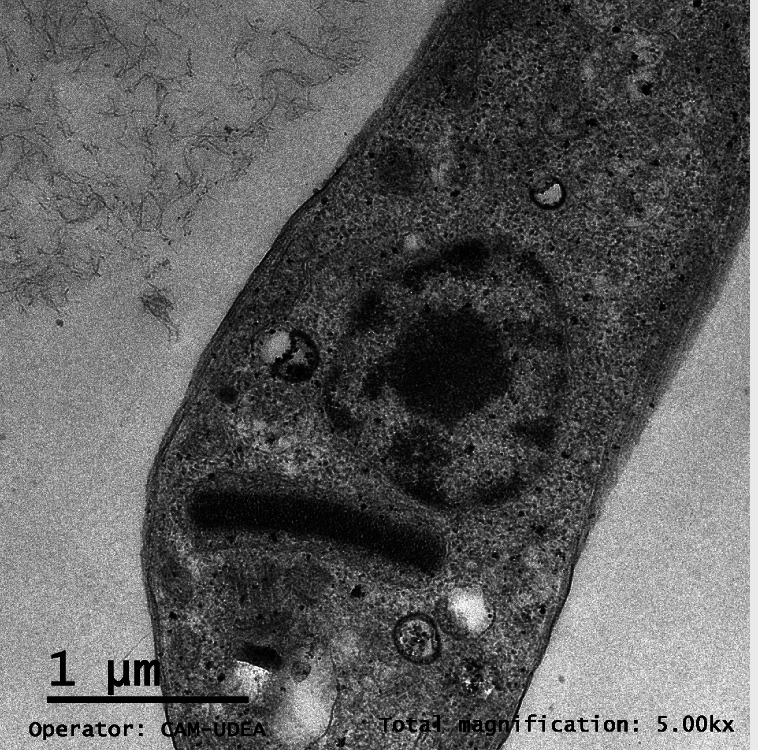


**figs2:**
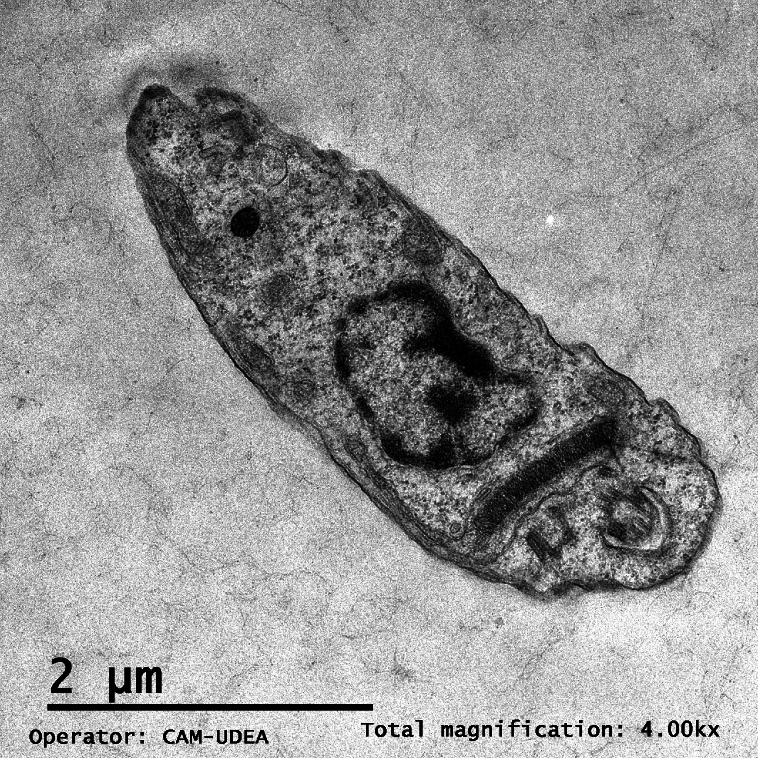


**figs3:**
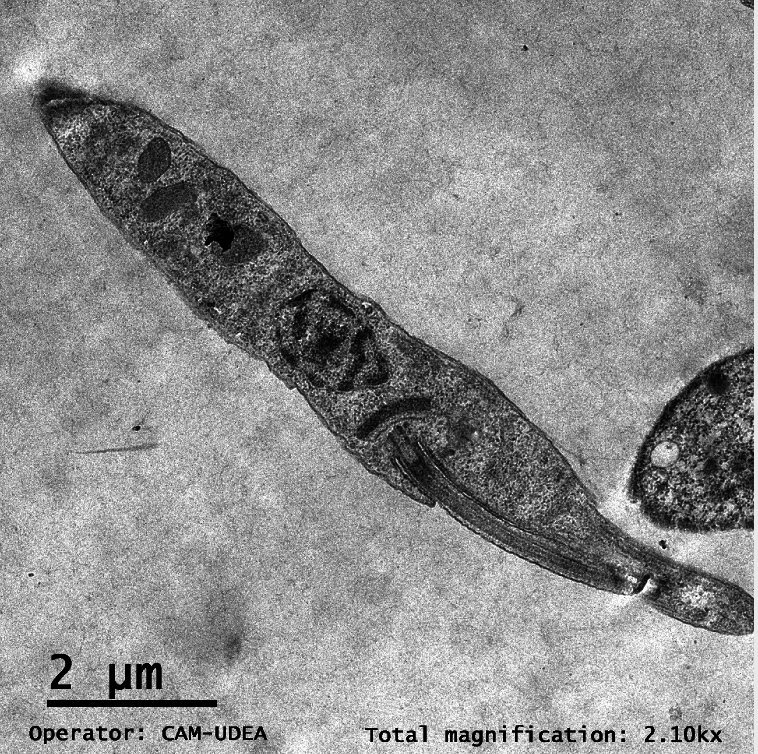


**figs4:**
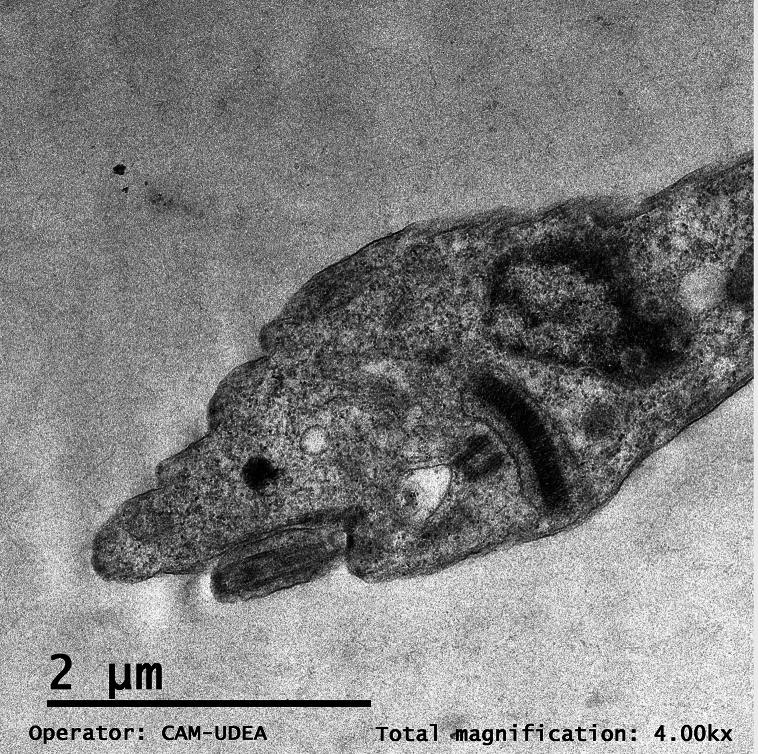


**figs5:**
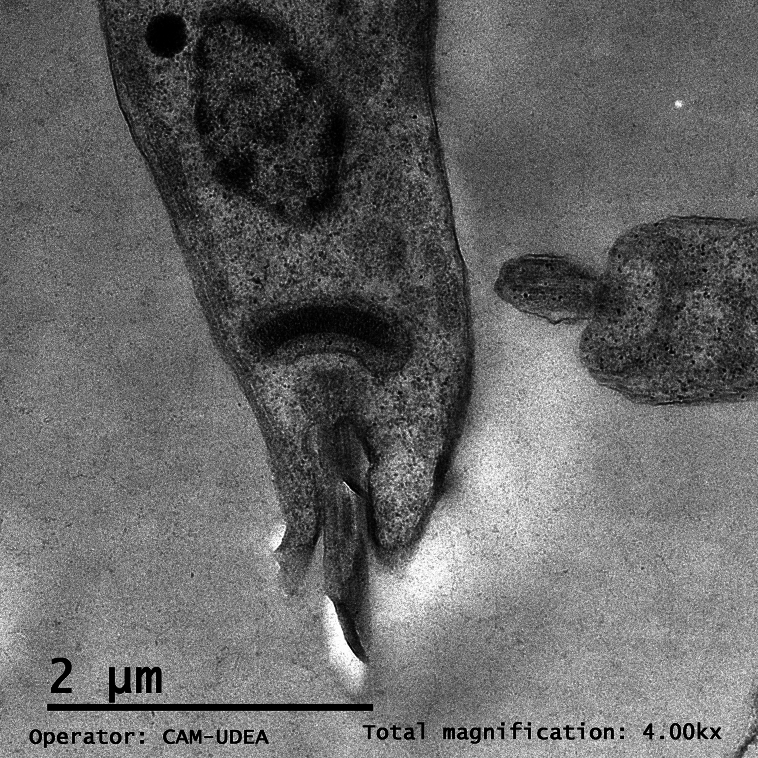


**figs6:**
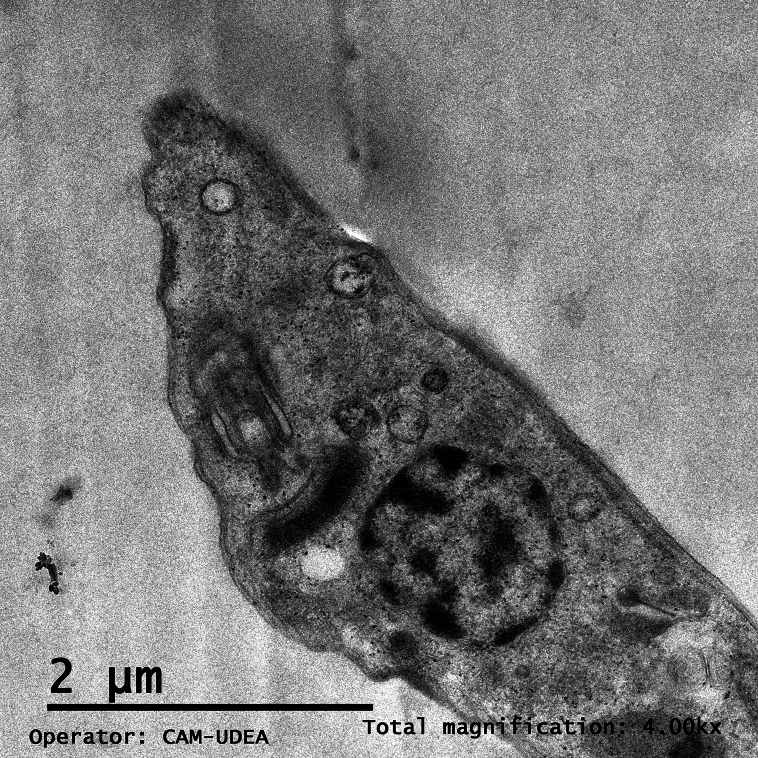


**figs7:**
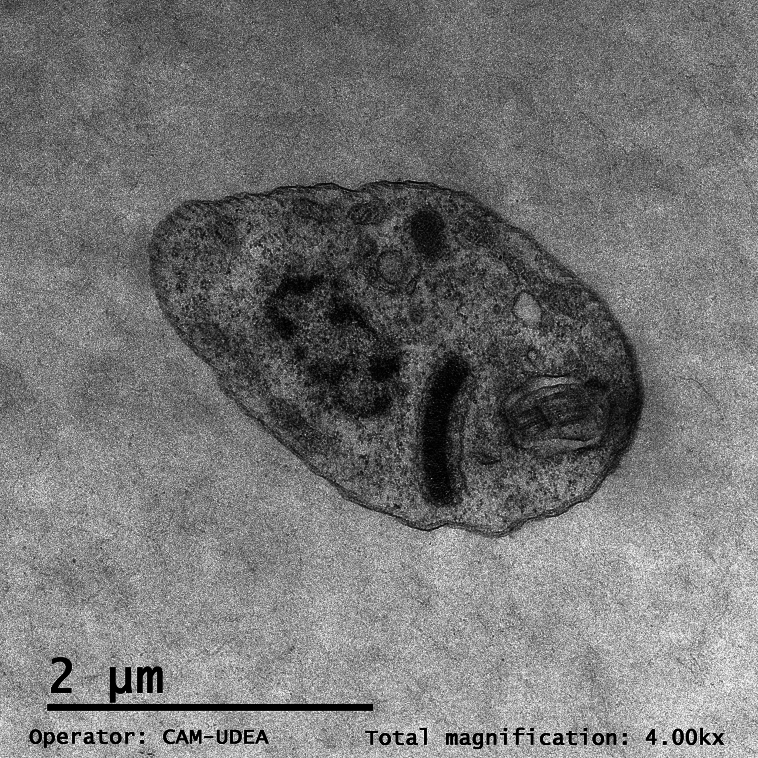


**figs8:**
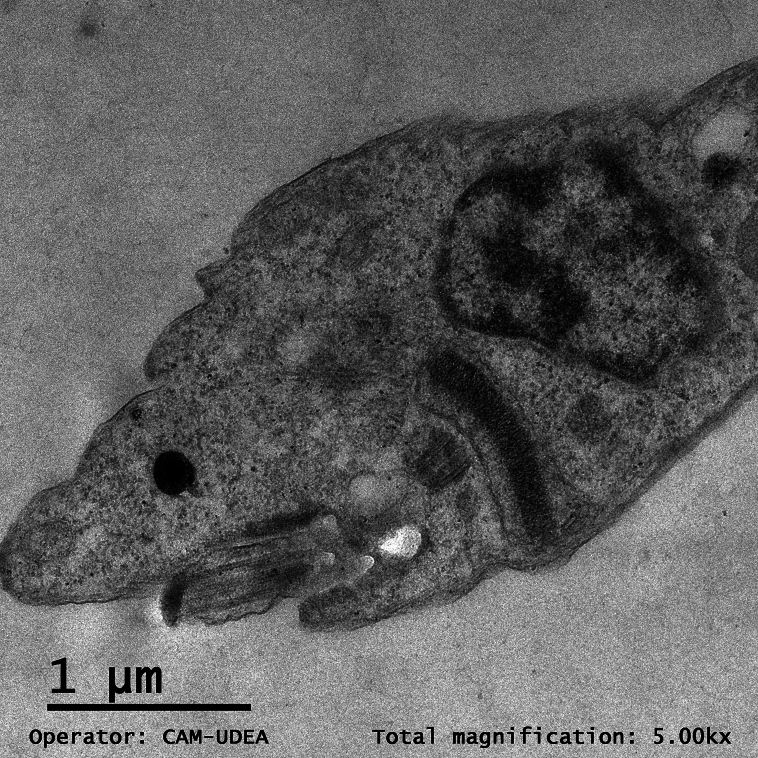


**figs9:**
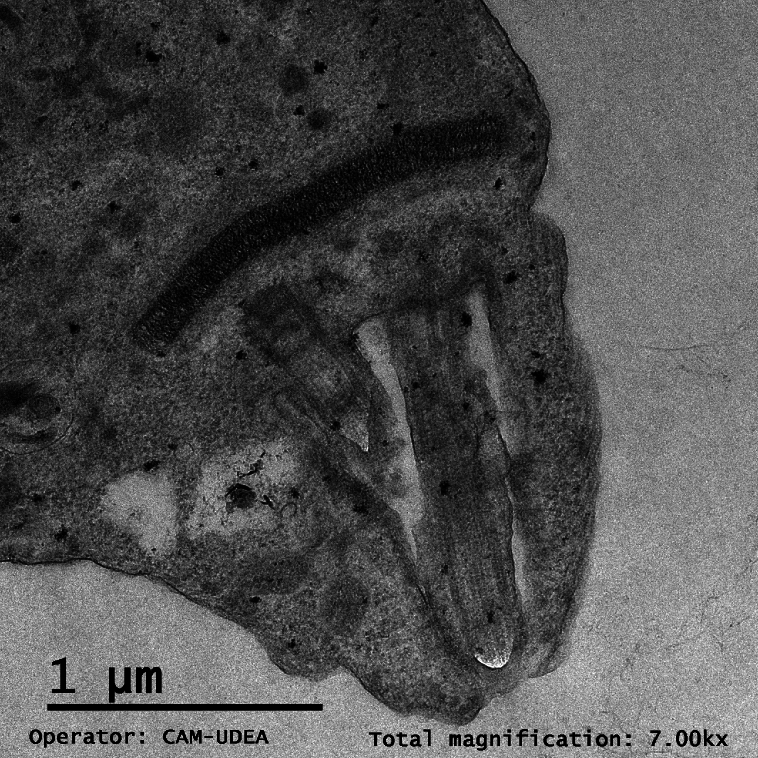


**figs10:**
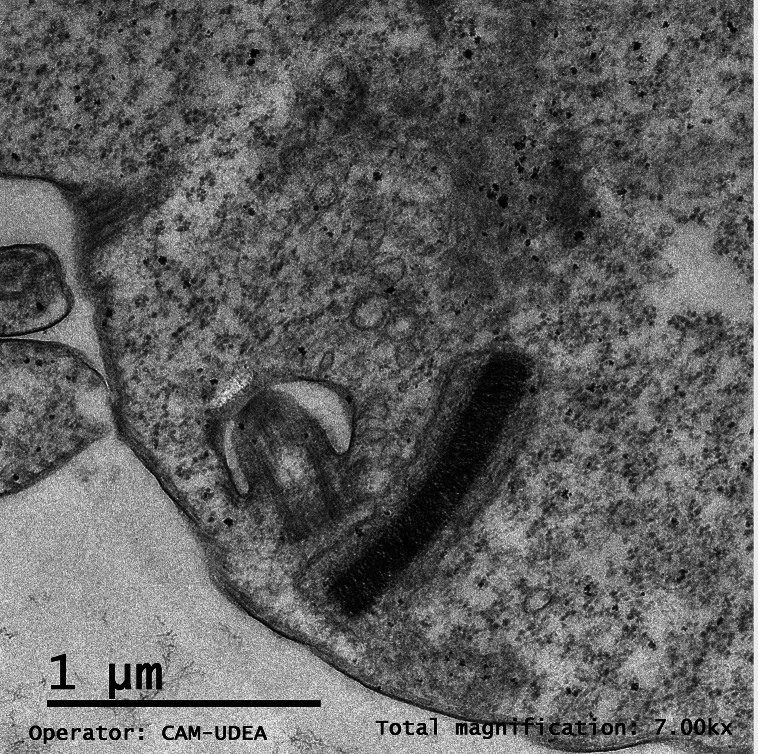


**figs11:**
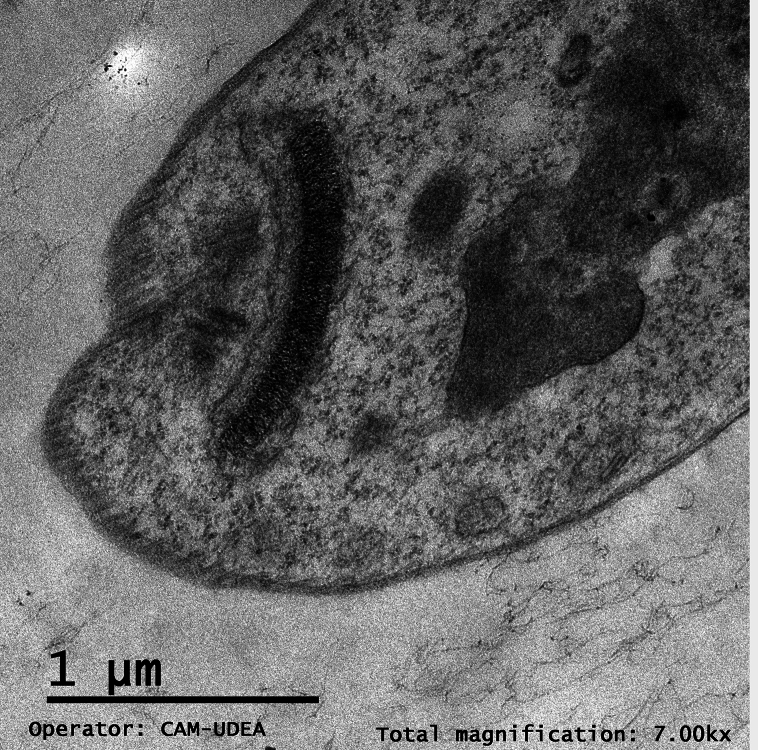


**figs12:**
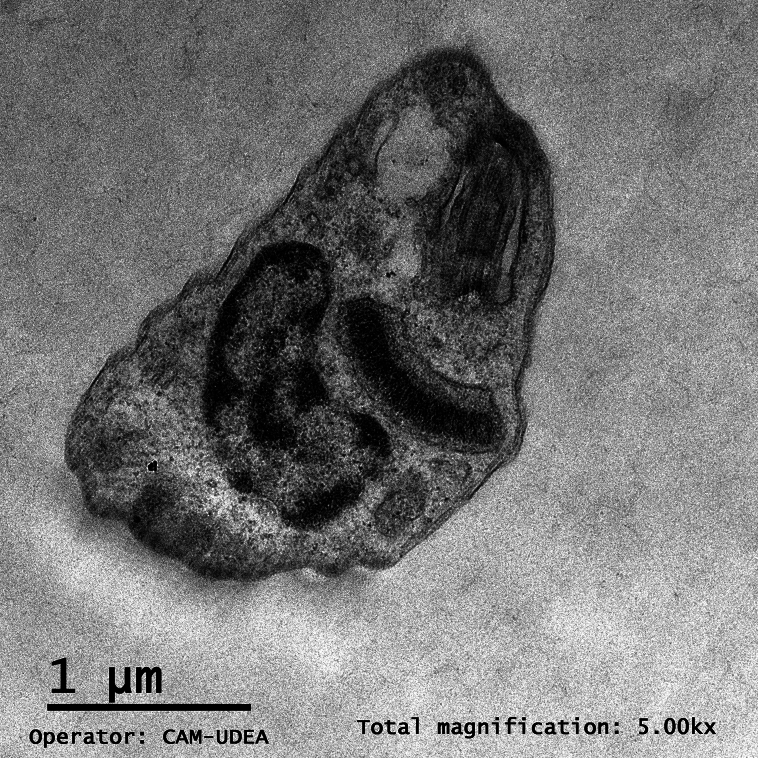


**figs13:**
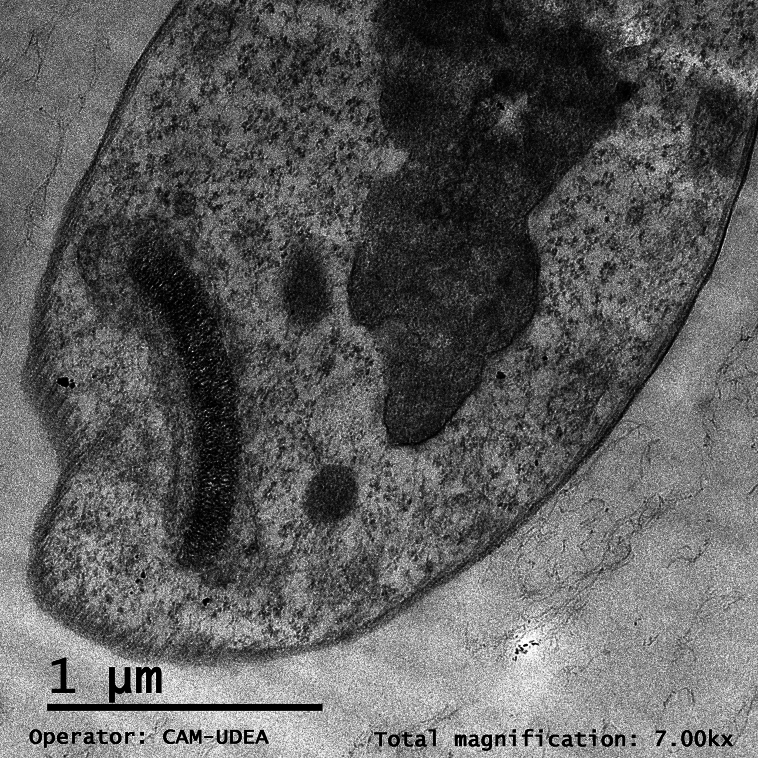


**figs14:**
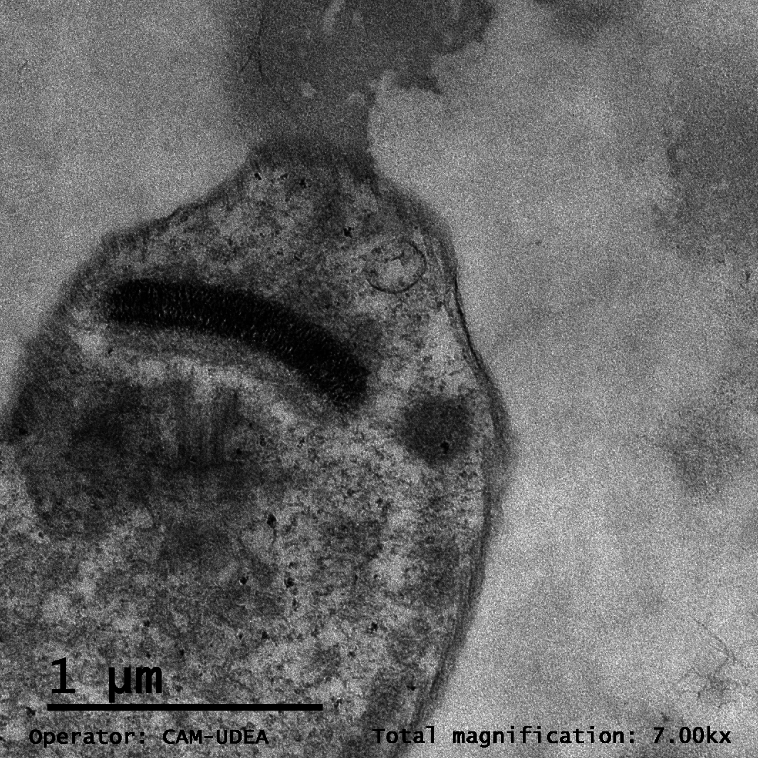


**figs15:**
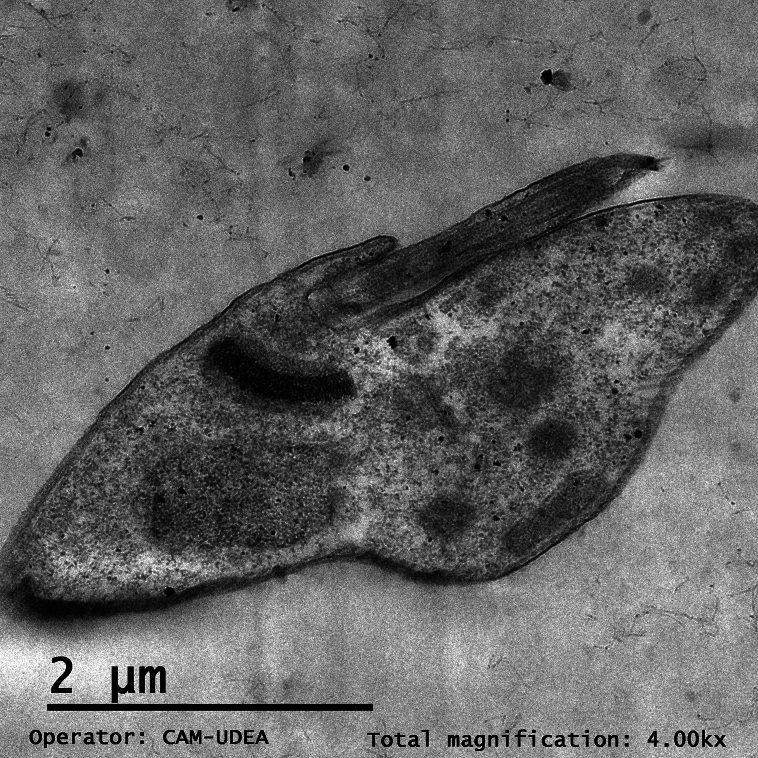


**figs16:**
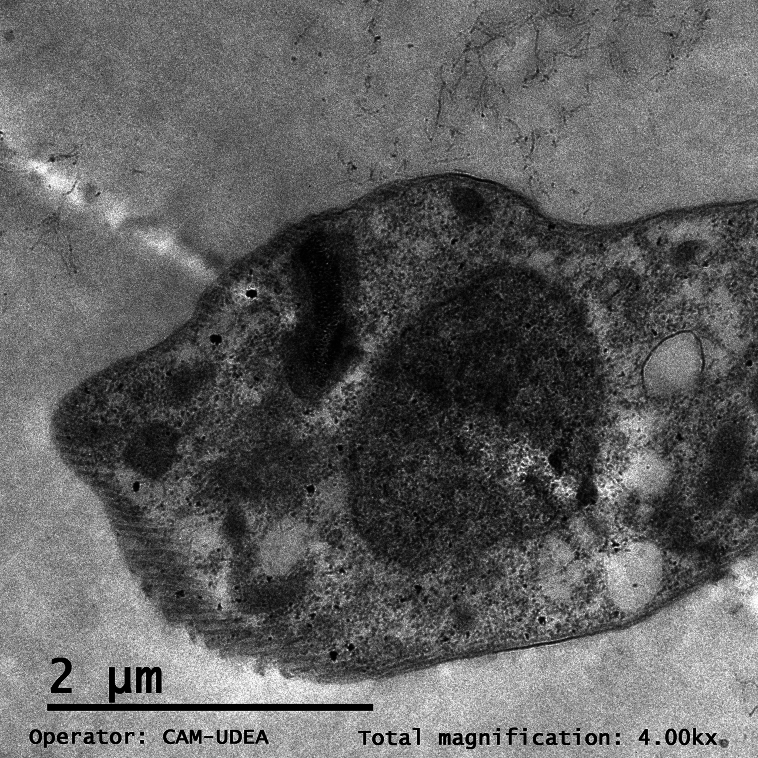


**figs17:**
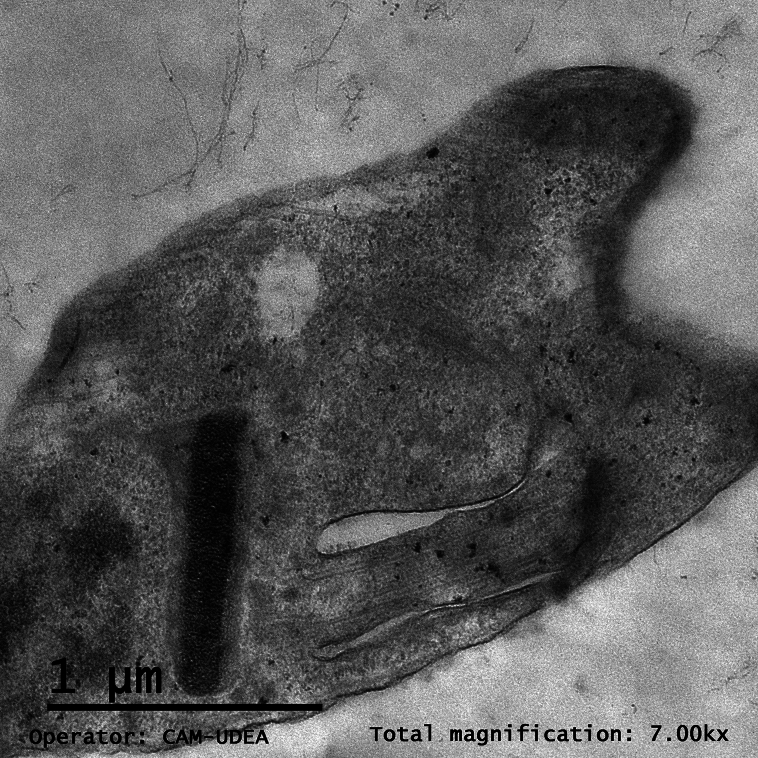


**figs18:**
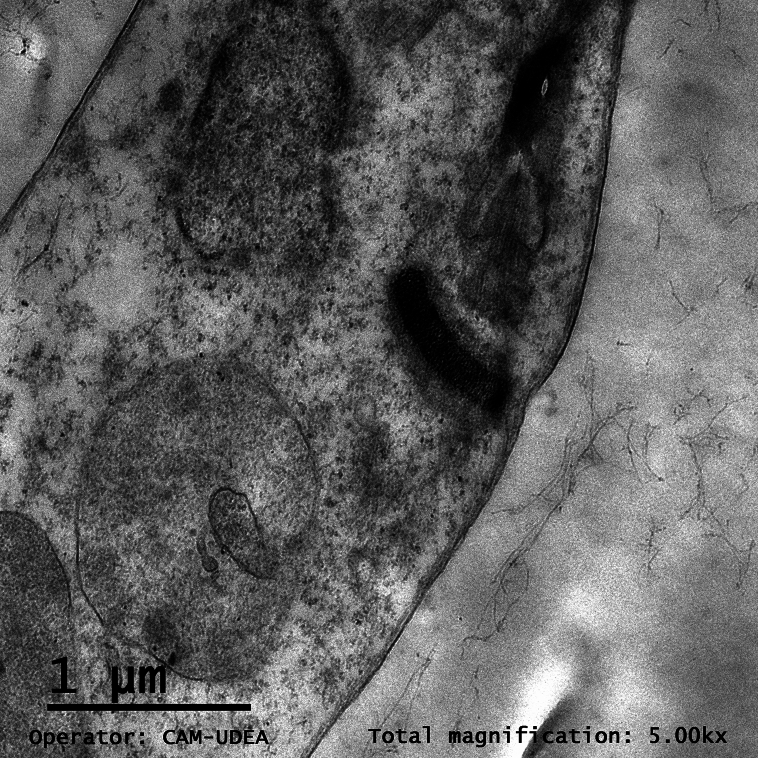


**figs19:**
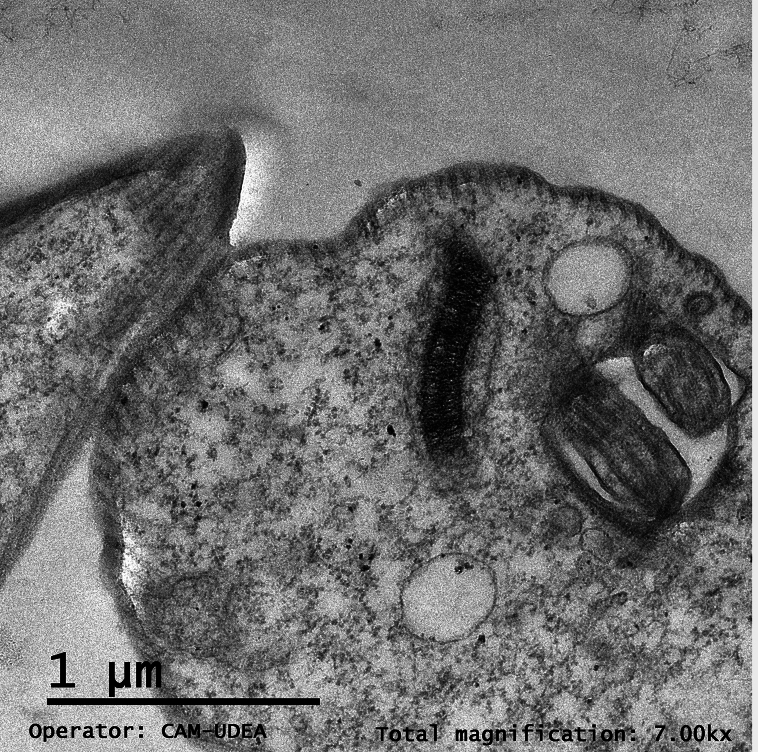


**figs20:**
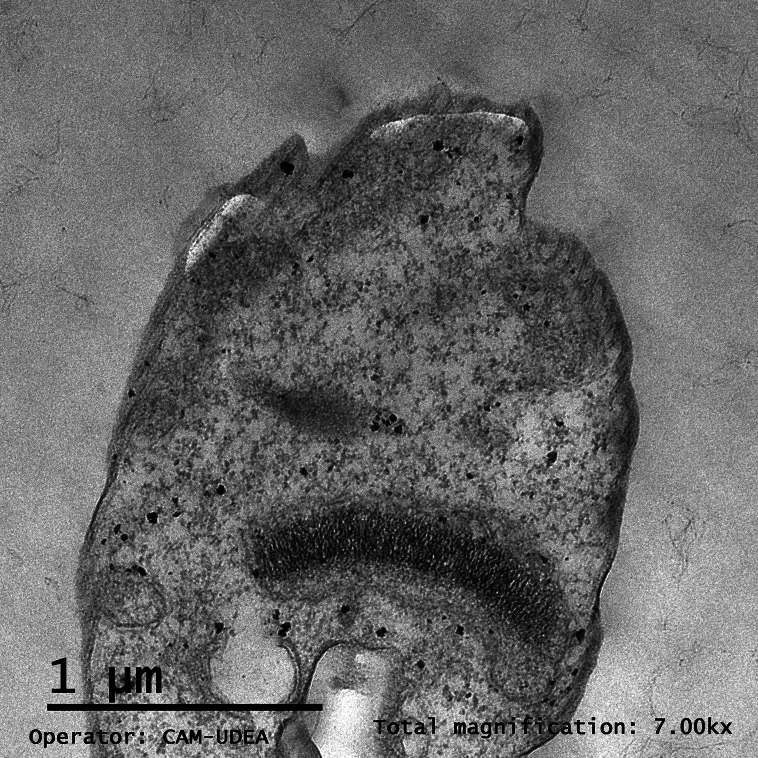


**figs21:**
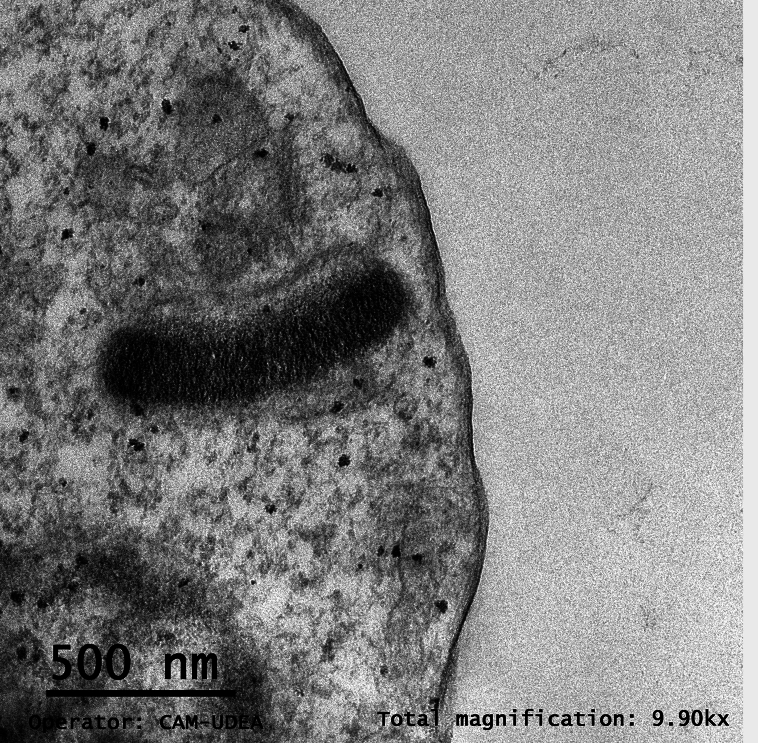


**figs22:**
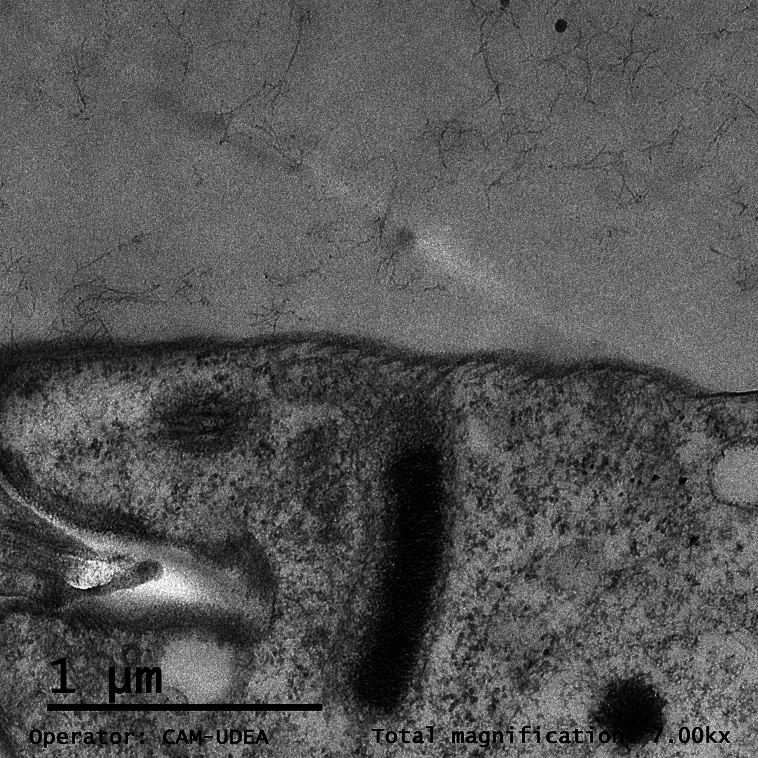


**figs23:**
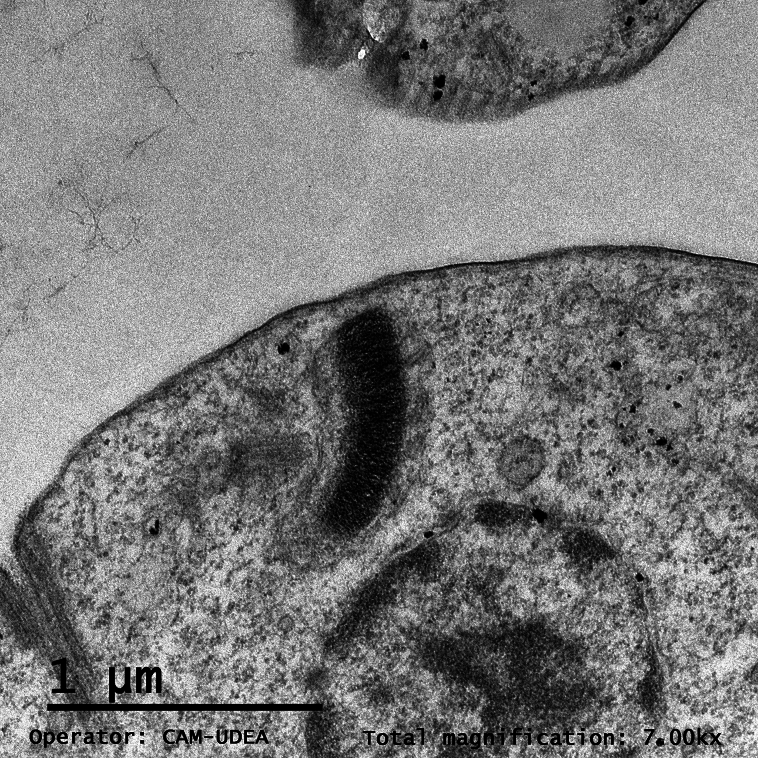


**figs24:**
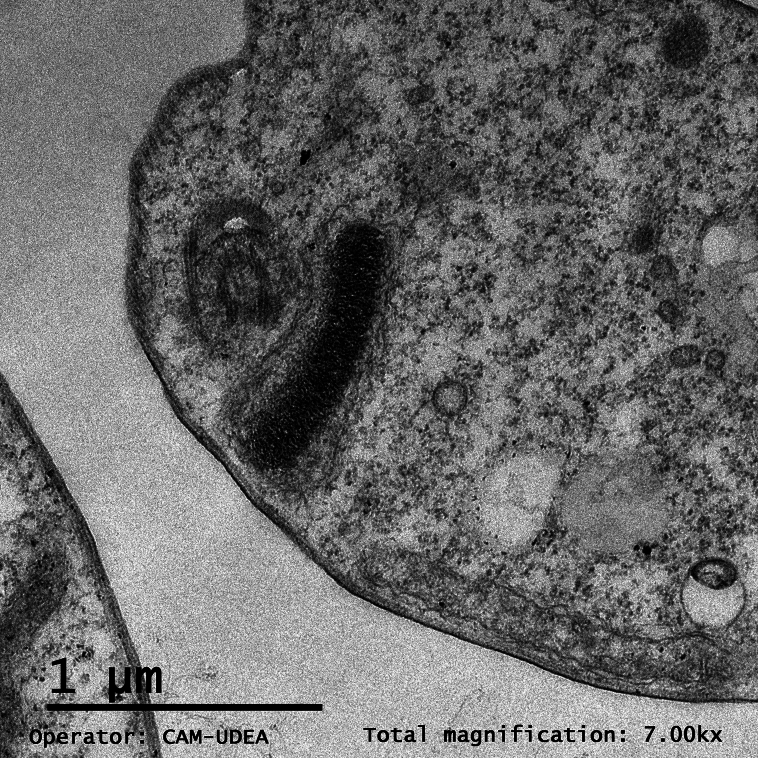


**figs25:**
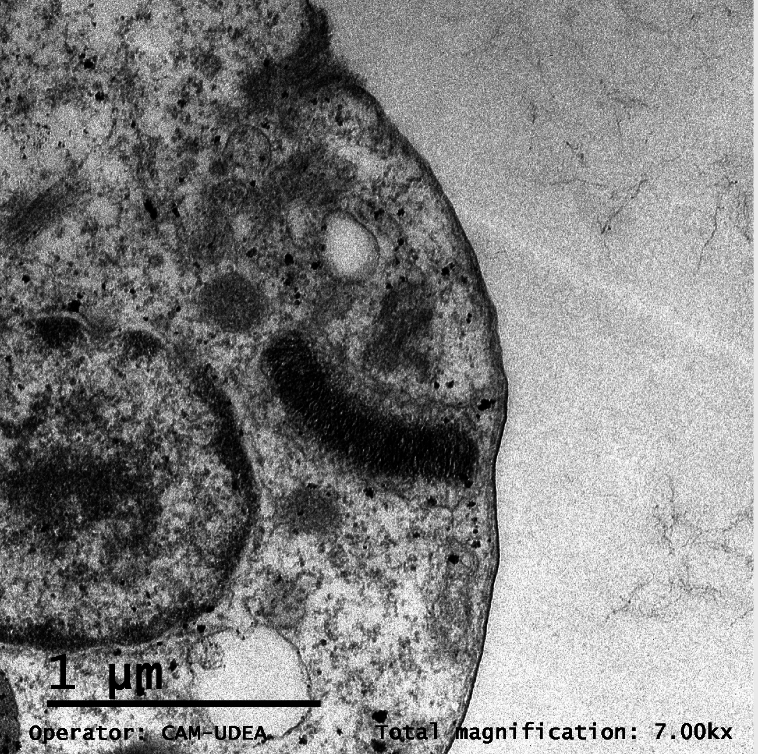


**figs26:**
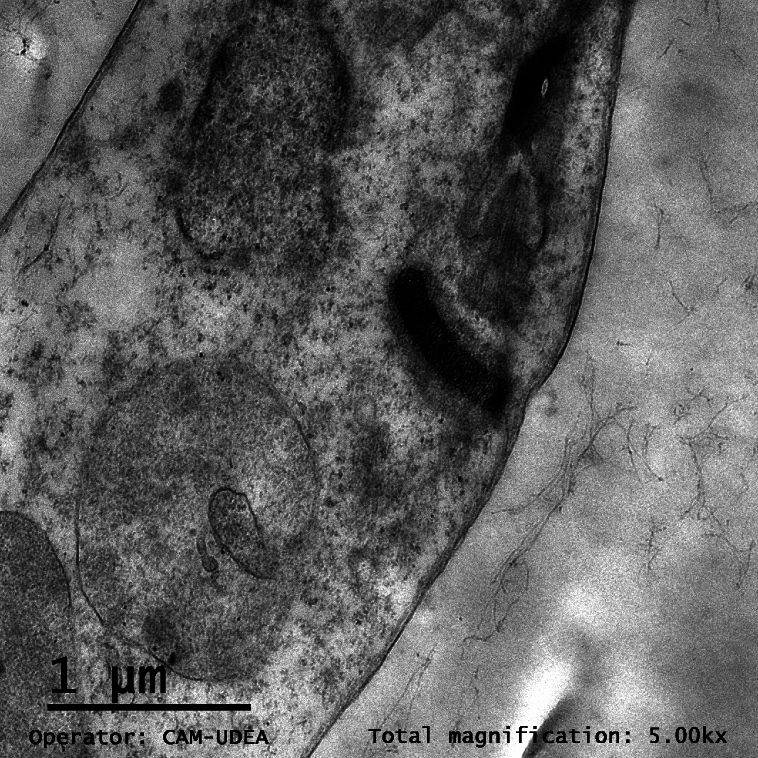


**figs27:**
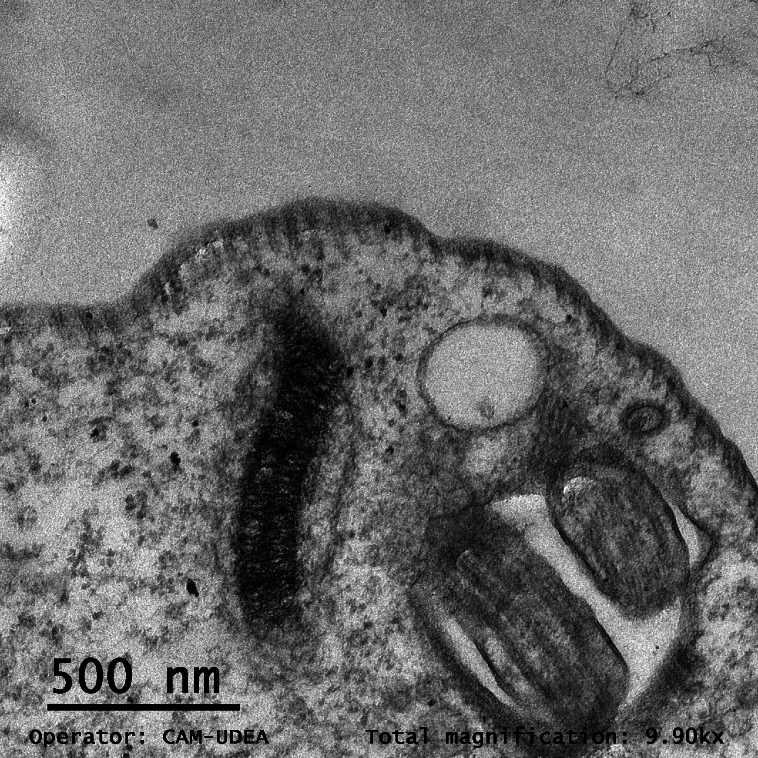


**figs28:**
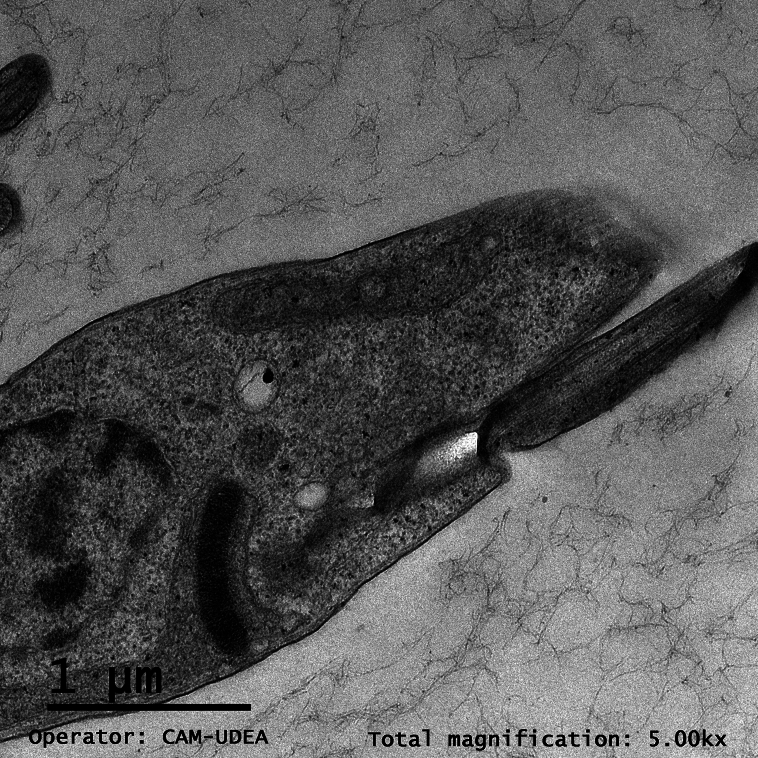


**figs29:**
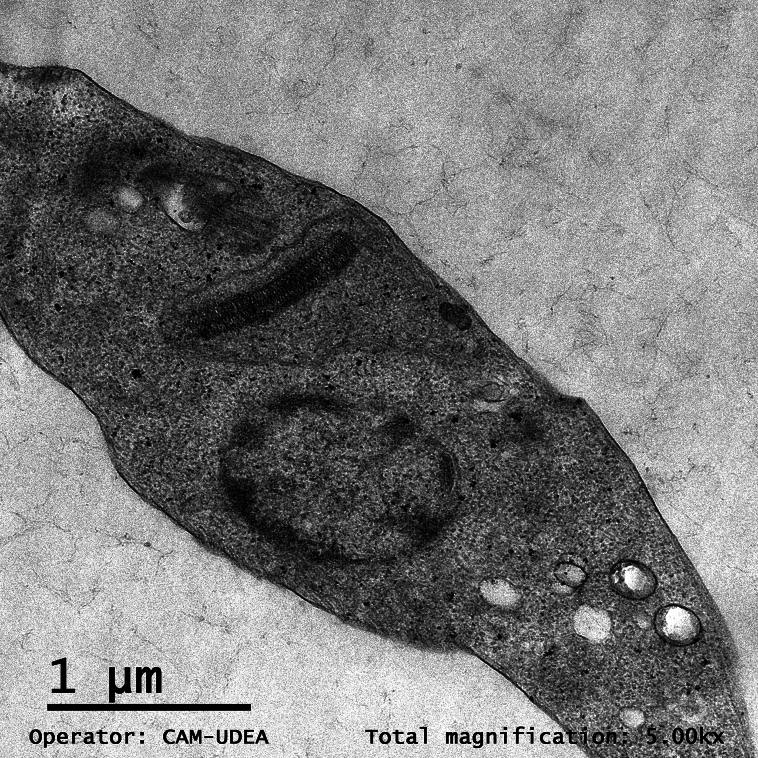


**figs30:**
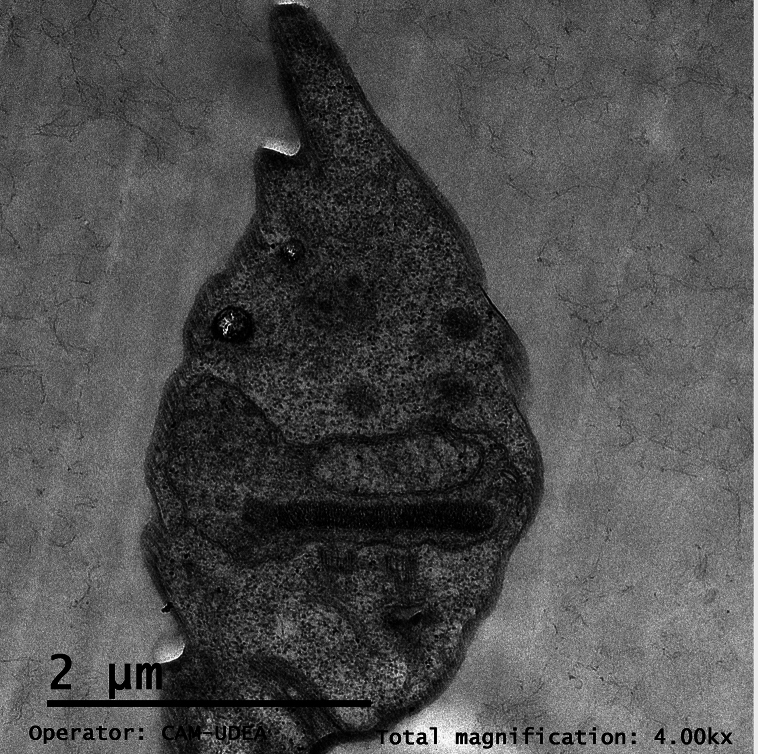


**figs31:**
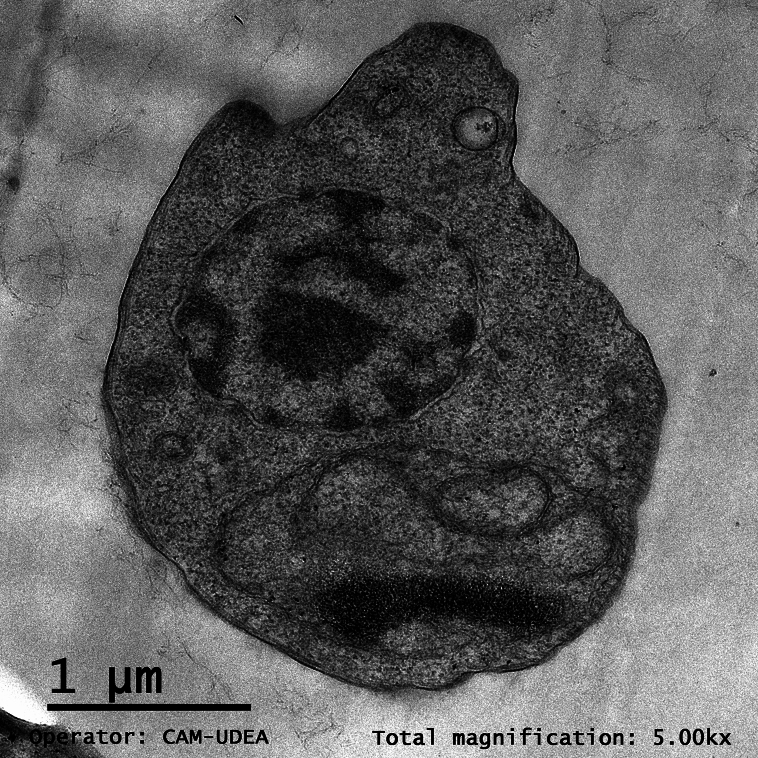


**figs32:**
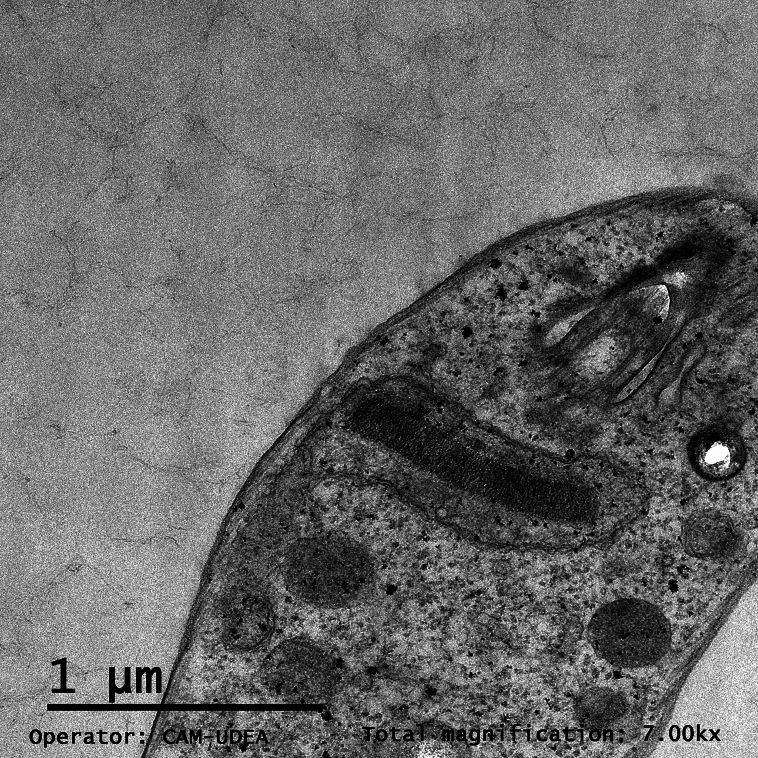


**figs33:**
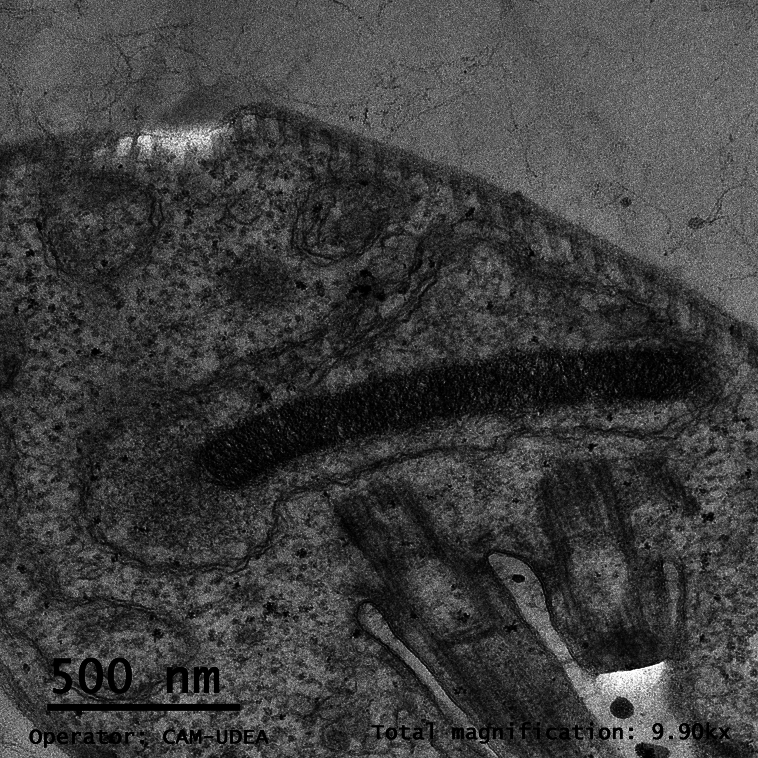


**figs34:**
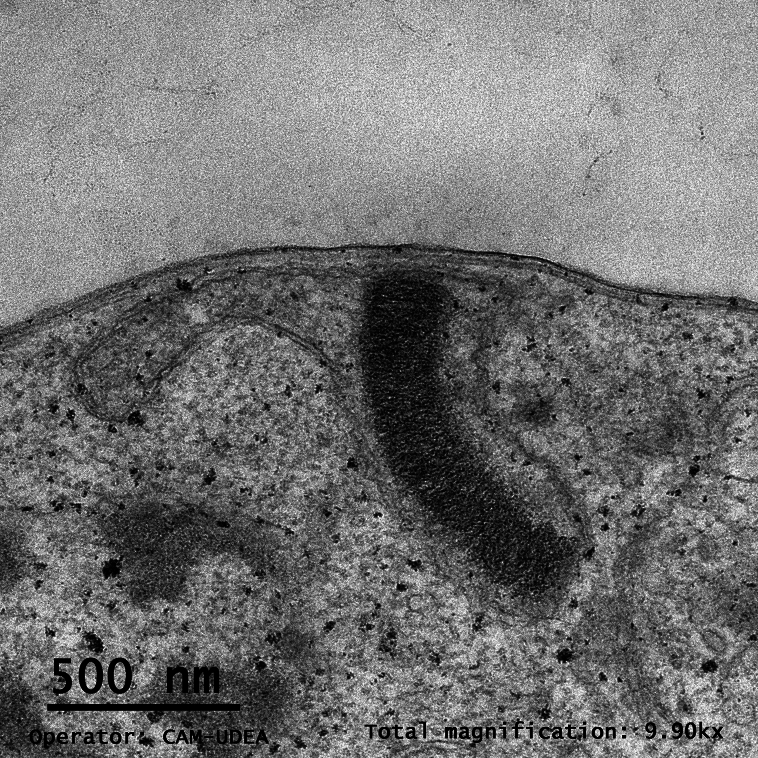


**figs35:**
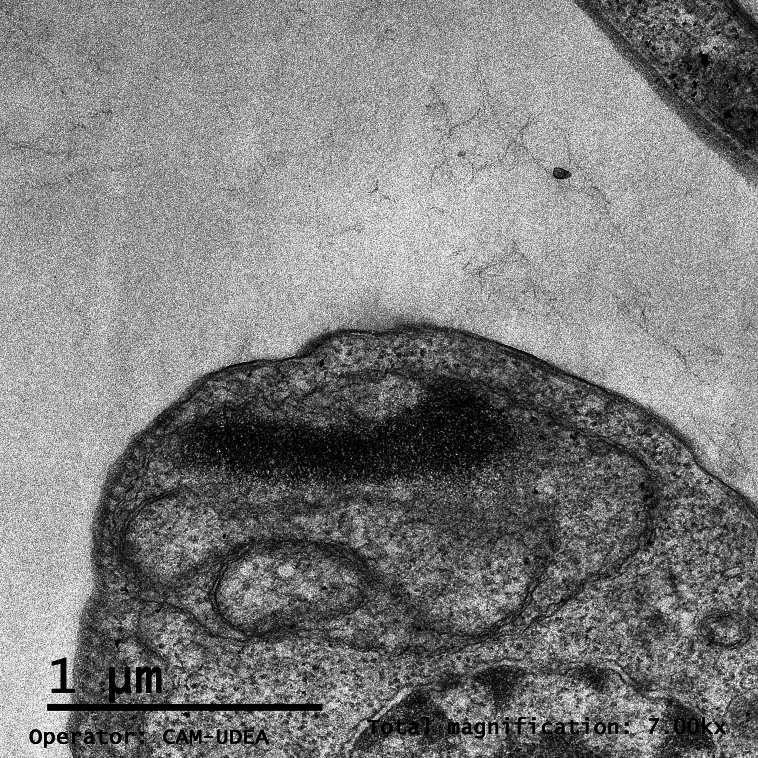


**figs36:**
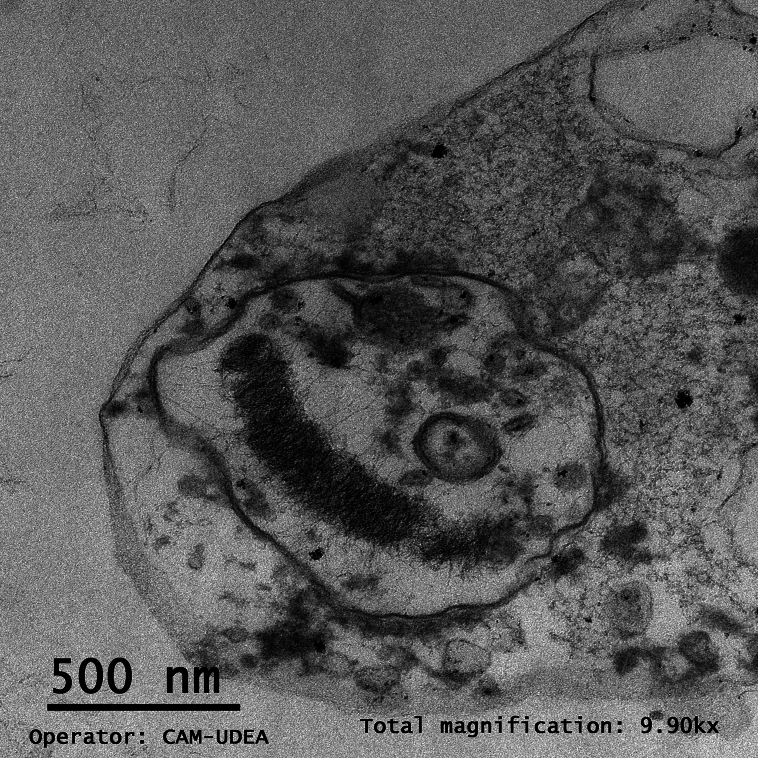


**figs37:**
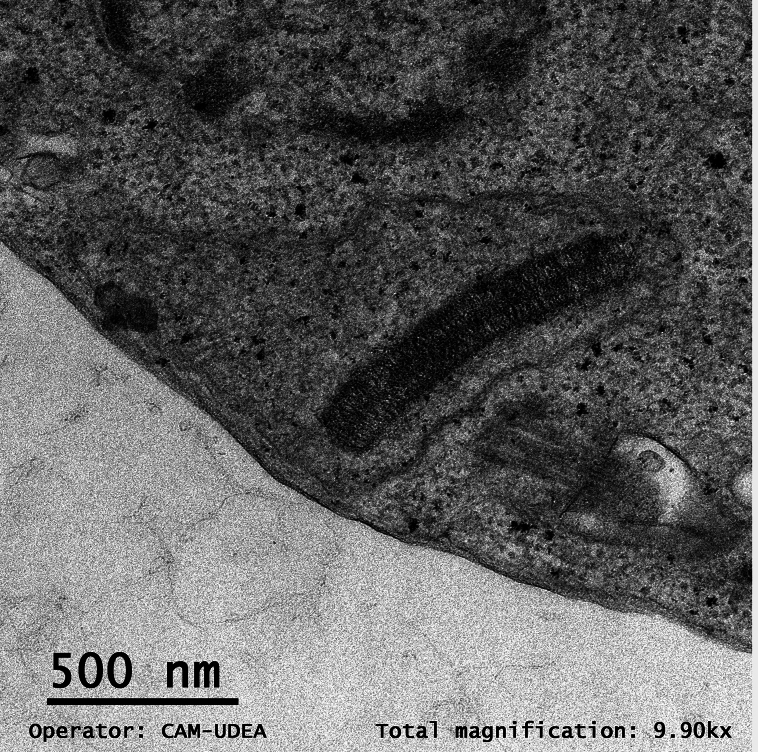


**figs38:**
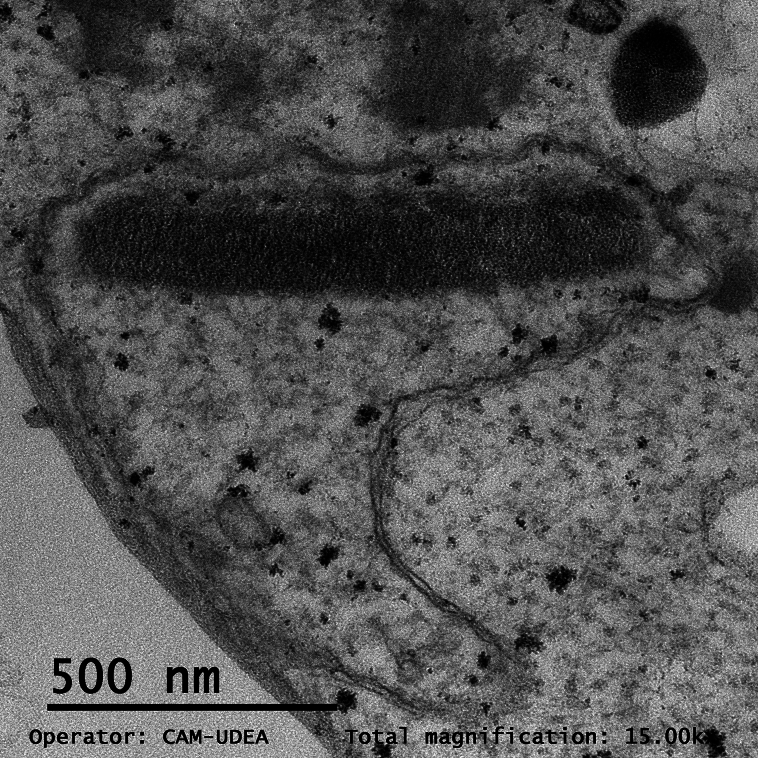


**figs39:**
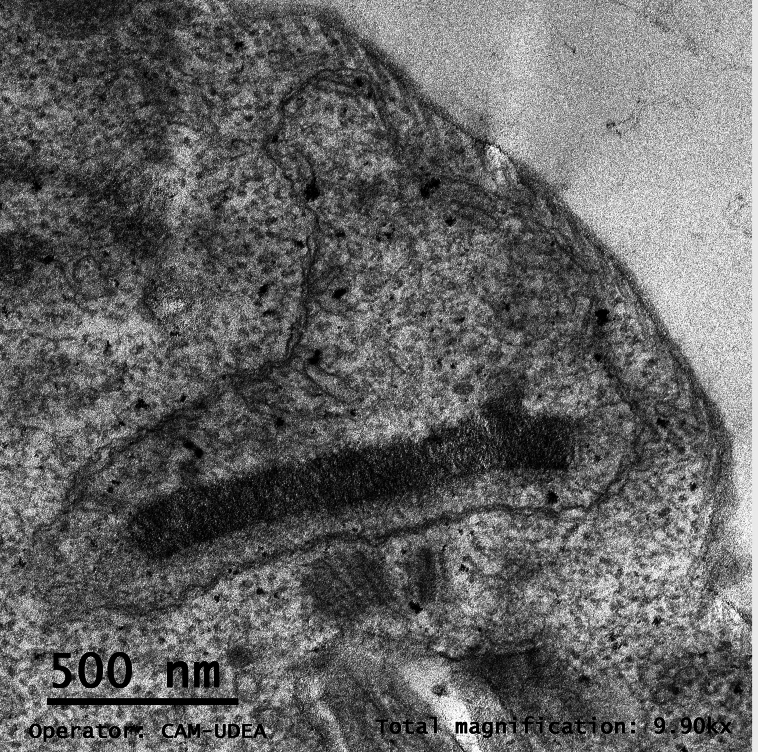


**figs40:**
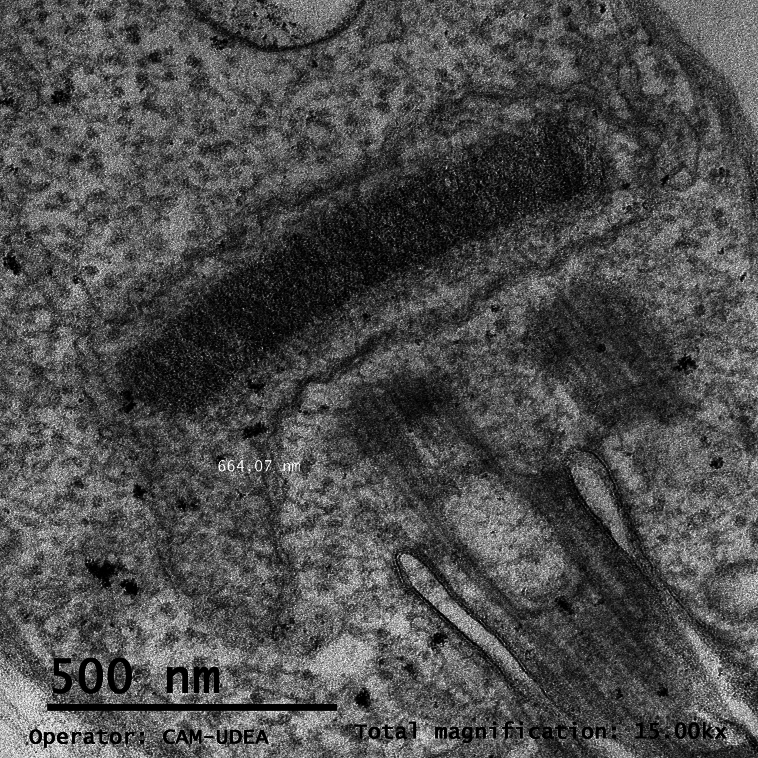


**figs41:**