# Supplementary material for: PUF3 RNA binding protein of Trypanosoma cruzi regulates mitochondrial morphology and function
Source: Heliyon. 2024 Jun 14;10(12):e32810. doi: 10.1016/j.heliyon.2024.e32810 (PMC11252720; doi:10.1016/j.heliyon.2024.e32810)
Supplement: Multimedia component 5 [file mmc5.docx]

**
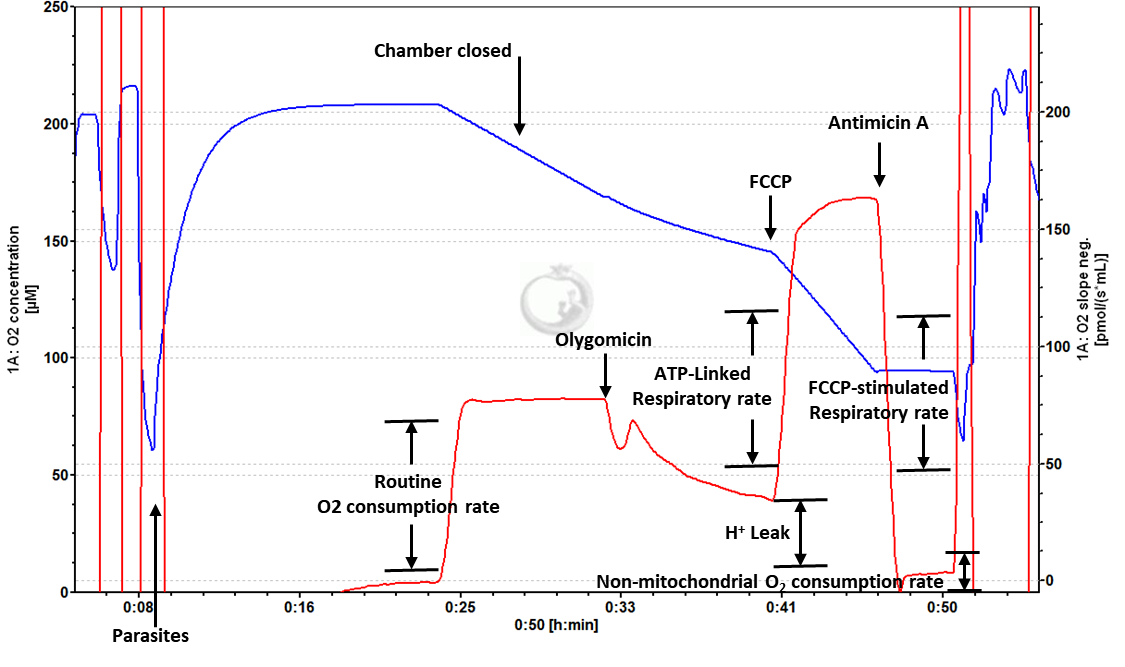
**

**Fig. S1**. **Scheme of oxygen consumption determined by oximetry.**

**
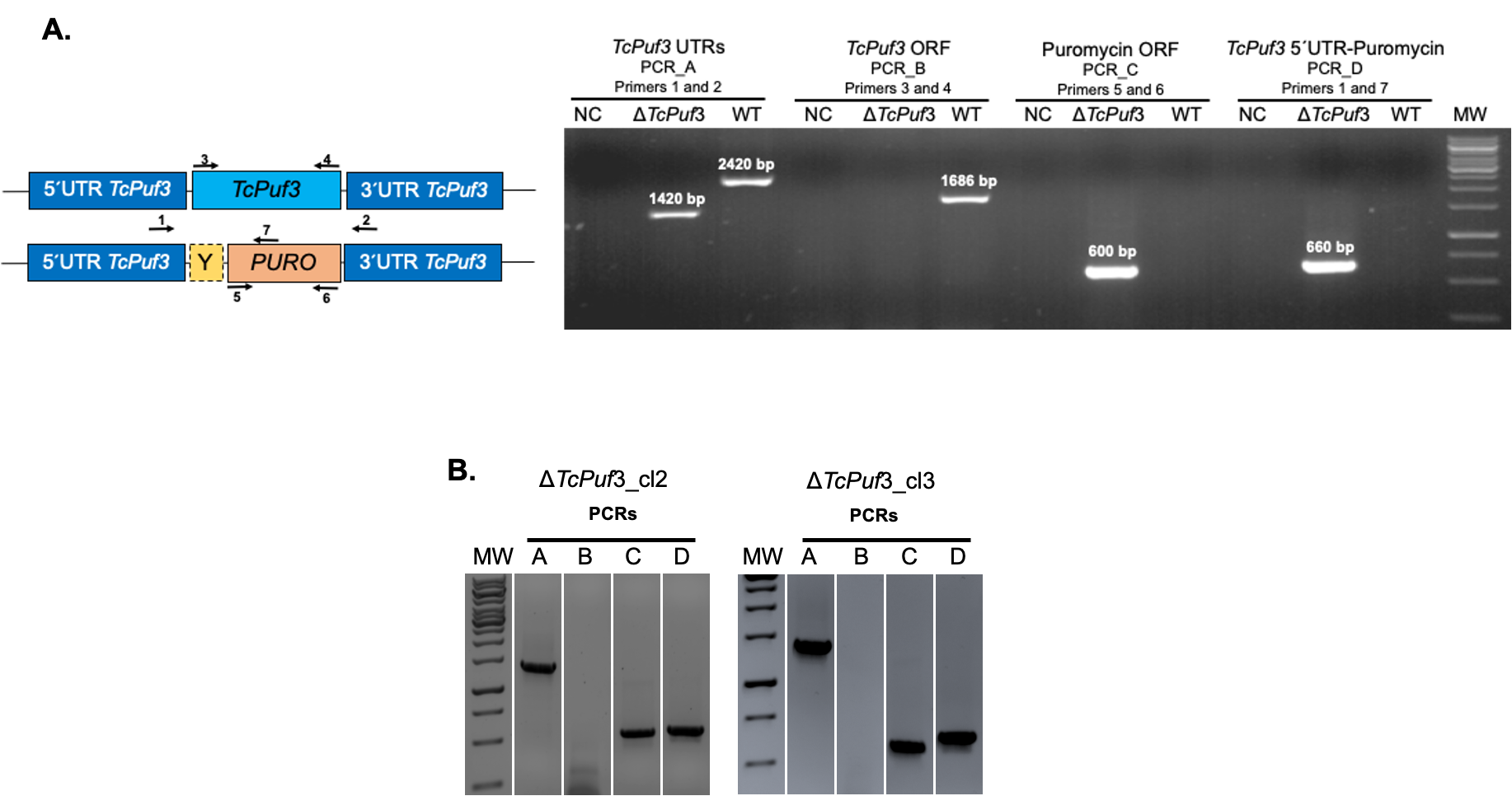
**

**Fig. S2**. **Confirmation of *Puf3* double knockout in *T. cruzi***. **A.** Schematic representations of different PCRs performed to confirm the double knockout of *Puf3* and the integration of the puromycin (PURO) gene in the *TcPuf3 l*ocus. The agarose gel shows the result of the PCR of control parasites (WT) and one clone (Δ*TcPuf3)* obtained after the transfection with RNP Cas9 complex and HRTs. **B.** Two additional knockout clones (Δ*TcPuf3*_cl2 and Δ*TcPuf3*_cl3) were also analyzed. Each letter (A, B, C, and D) corresponds to PCRs from Figure A. All the primer sequences are listed in the supplementary Table S1.


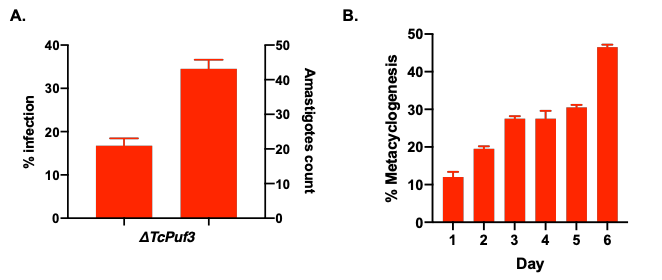


**Fig. S3**. **Differentiation of epimastigotes from knockout parasites to other life cycle stages. A.** *In vitro* infection in VERO cells and amastigotes count after 48 h. **B.** *In vitro* metacyclogenesis. The parasites were maintained in the TAU 3AAG differentiation medium and counted every 24 h for six days.


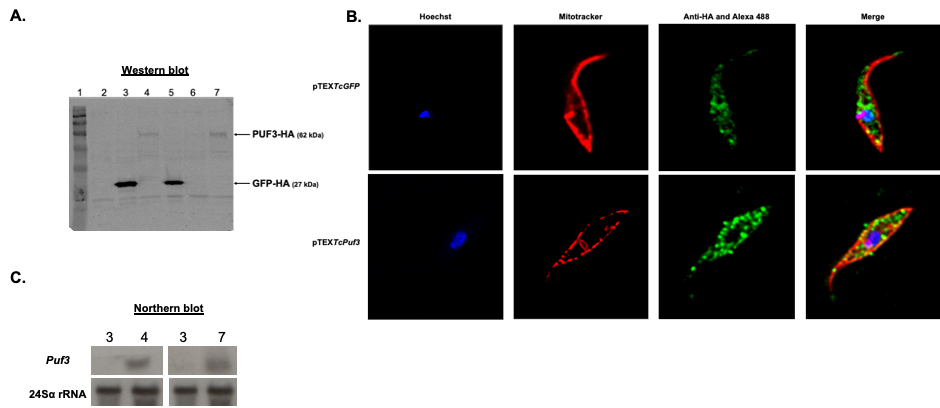


**Fig. S4**. **Localization and overexpression of PUF3 in *T. cruzi***. **A.** Western blot HA-tagged proteins from *T. cruzi*. 1, protein ladder; 2, WT parasites; 3, HA-tagged pTEX*GFP*; 4, HA-tagged pTEX*TcPuf3* parasites; 5, Δ*TcPuf3* transfected with pTREX*GFP*-HA; 6, Δ*TcPuf3* parasites; 7, Δ*TcPuf3* transfected with pTREX*TcPuf3*. One hundred μg of proteins were load in each line. Anti-HA (1:1000) was used as the primary antibody, and the IRDye 800 (1:15,000) CW Donkey anti-rabbit was secondary. **B.** Immunofluorescence microscopy localization using HA-tagged PUF3 and GFP proteins in *T. cruzi*. The parasites were incubated with MitoTracker™ (red), and as a primary antibody, anti-HA was used and as secondary anti-Rabbit Alexa 488 (green), with Hoechst 333342 (blue). The slides were analyzed on the Fluoview™, 1000, OLYMPUS® confocal microscope and the images were processed with the Fiji software (https://fiji.sc). **C.** Northern blot of *Puf3* overexpressing parasites compared with parasites transfected with GFP. The ribosomal 24S gene was used as the normalizer. The line numbers correspond to the ones in the Western blot.


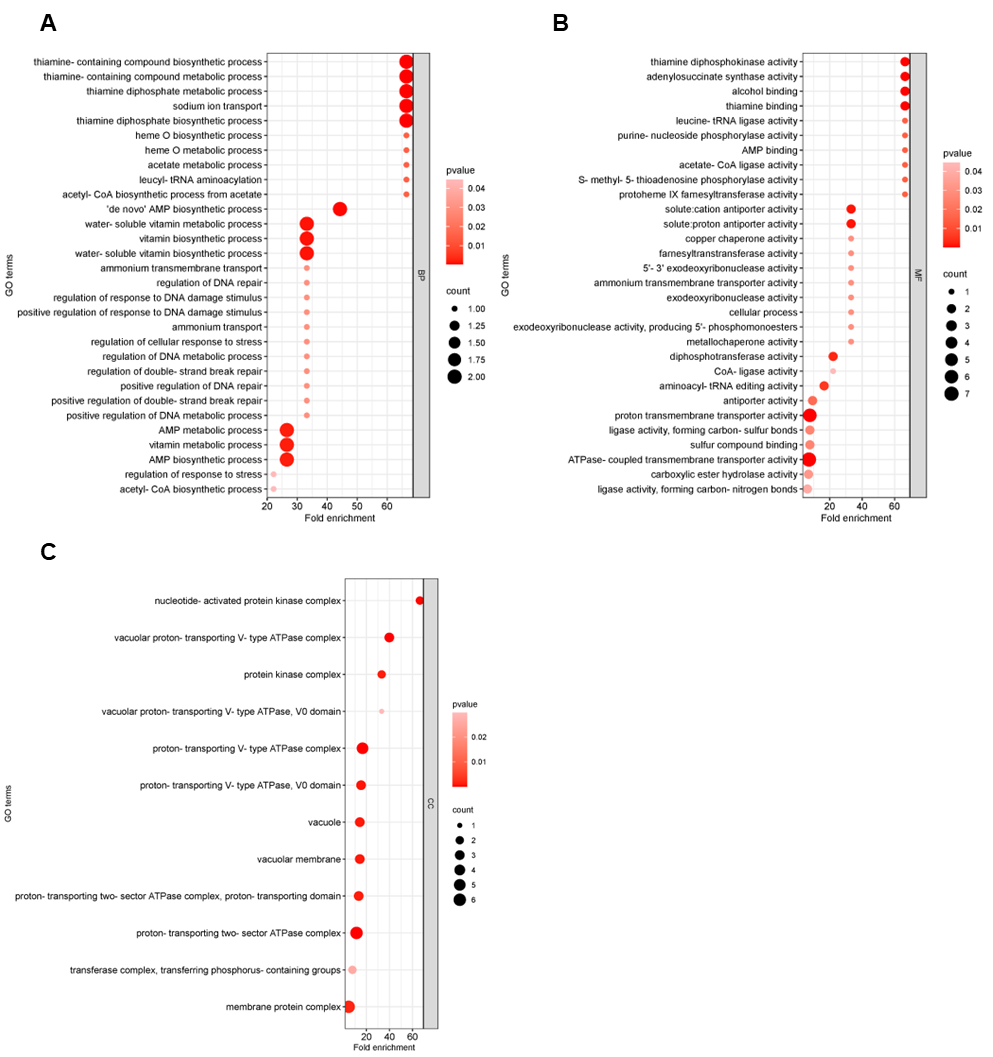


**Fig. S5**. **Gene ontology enrichment of differentially expressed genes in Δ*TcPuf3* parasites.** The graphs show the 30 GO terms with the highest fold enrichment for each domain except for cellular component which was plotted entirely (12 terms). The size of the circles indicates the number of differentially expressed genes, the intensity of their color refers to the p value, and their location on the X axis represents the fold enrichment. **A.** Biological processes. **B.** Molecular function. **C.** Cellular component.


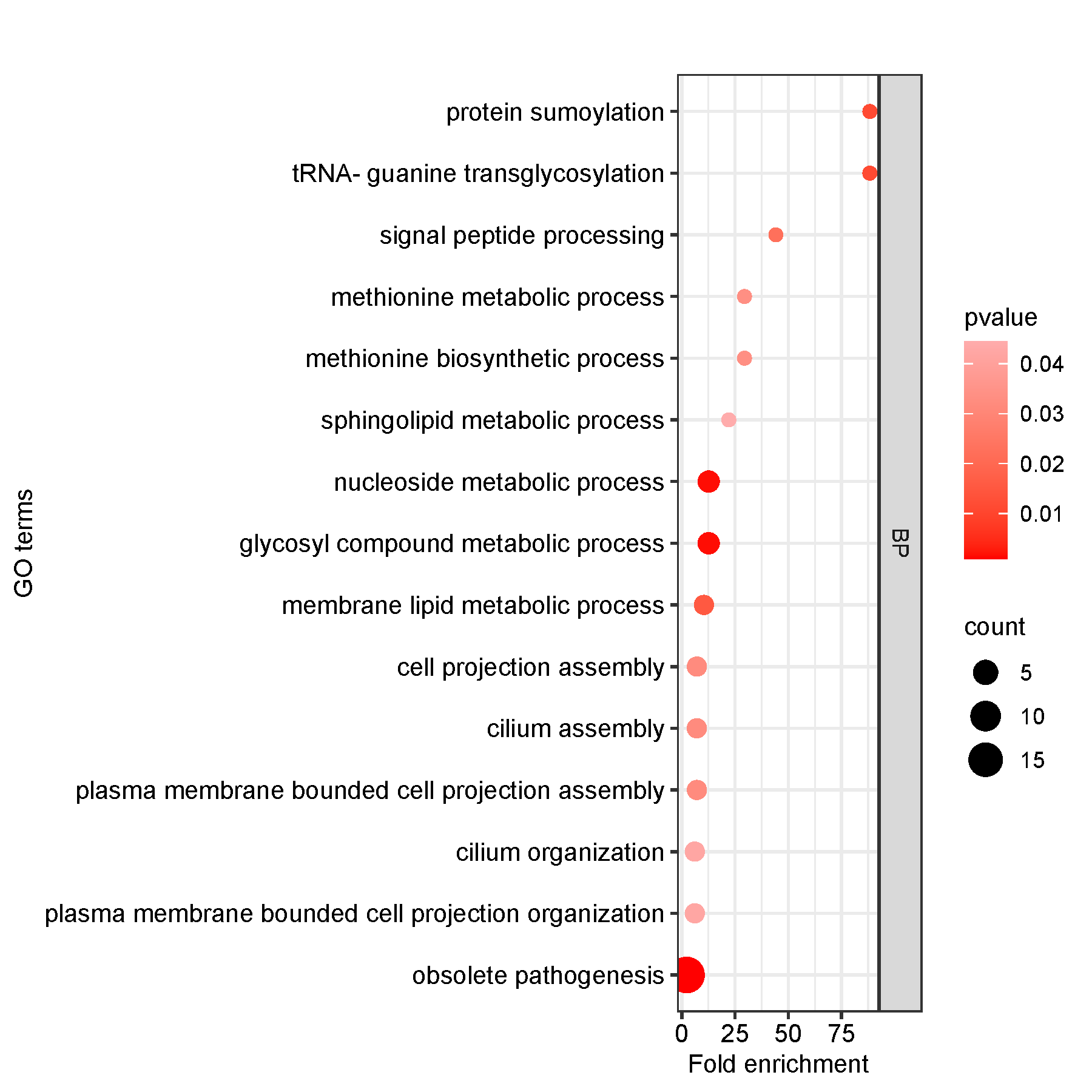

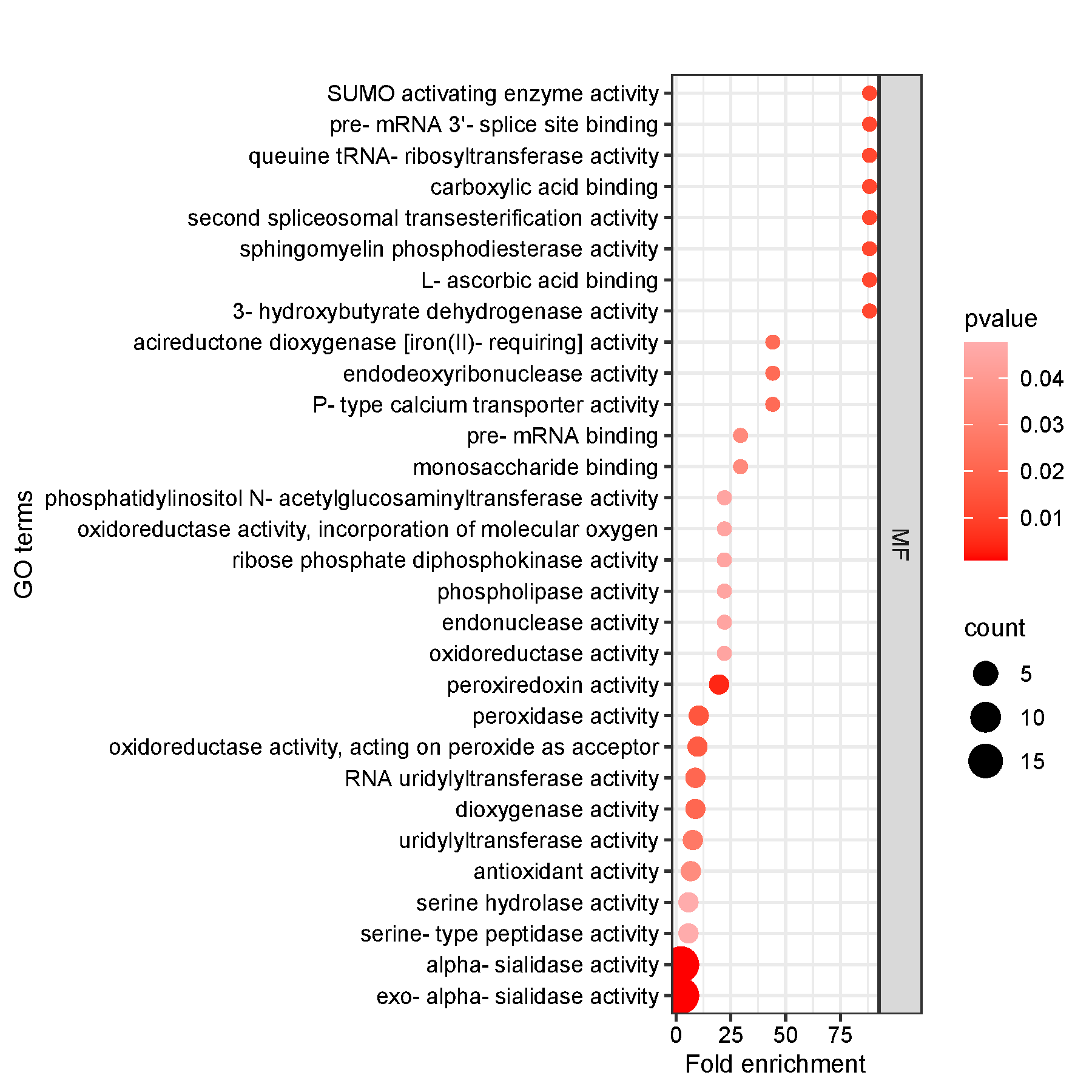

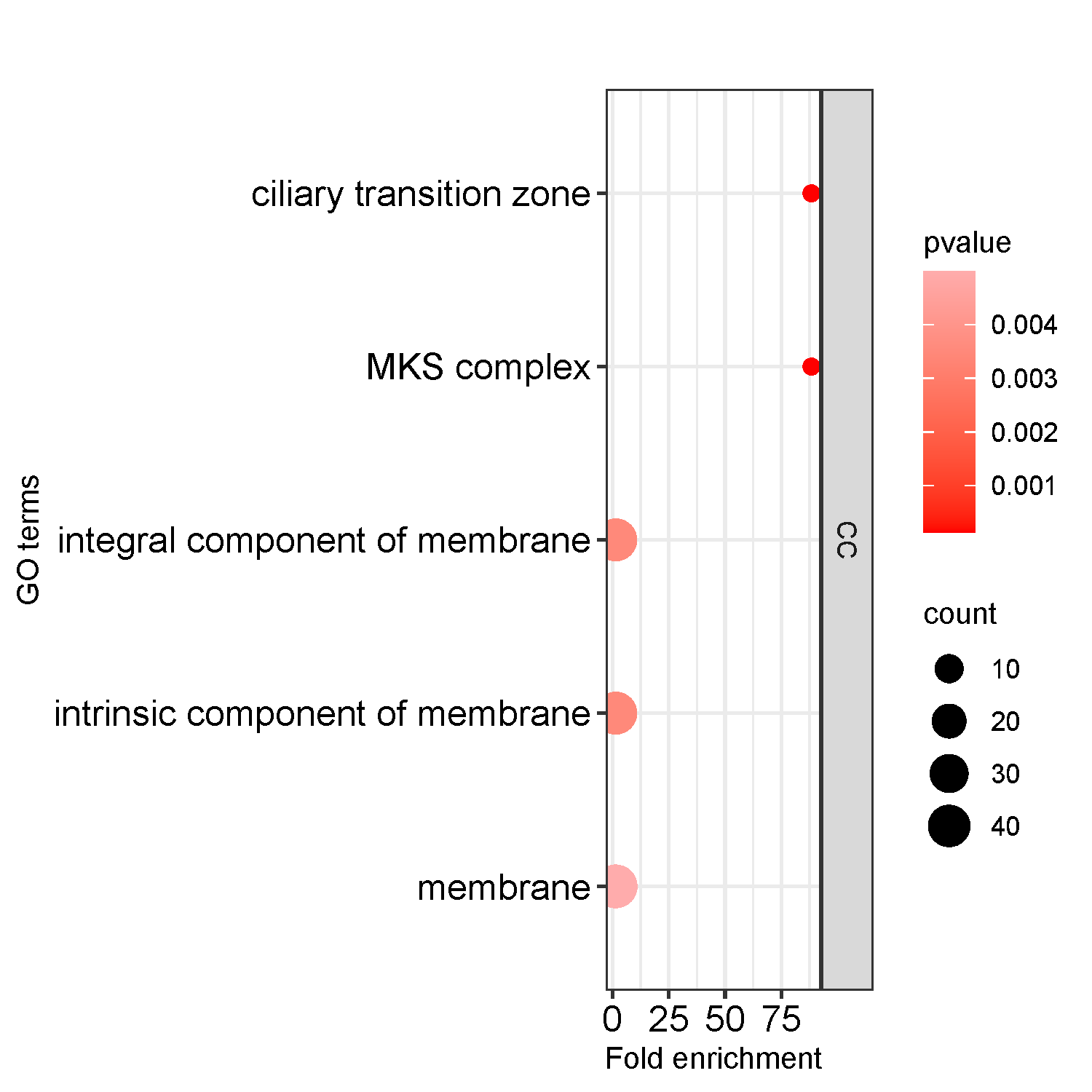


**A**

**B**

**C**

**Fig. S6**. **Gene ontology enrichment of differentially expressed genes in *pTEXTcPuf3* cells.** The graphs show a maximum 30 GO terms with the highest fold enrichment for molecular function, 15 GO terms for biological processes and 5 GO terms cellular component which were plotted entirely. The size of the circles indicates the number of differentially expressed genes, the intensity of their color refers to the p value, and their location on the X axis represents the fold enrichment. **A.** Biological processes. **B.** Molecular function. **C.** Cellular component.


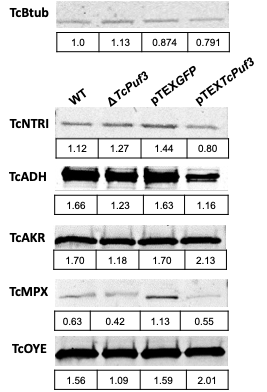


**Figure S7**. **Relative expression levels in *T. cruzi* proteins from TcPUF3 knockout and overexpressor populations**. Expression levels of different proteins in WT, pTEX*GFP*, Δ*TcPuf3* and pTEX*TcPuf3* populations were determined by WB. The intensity of the signals was normalized with those obtained for β-tubulin. The bands were quantified using ImageJ software.


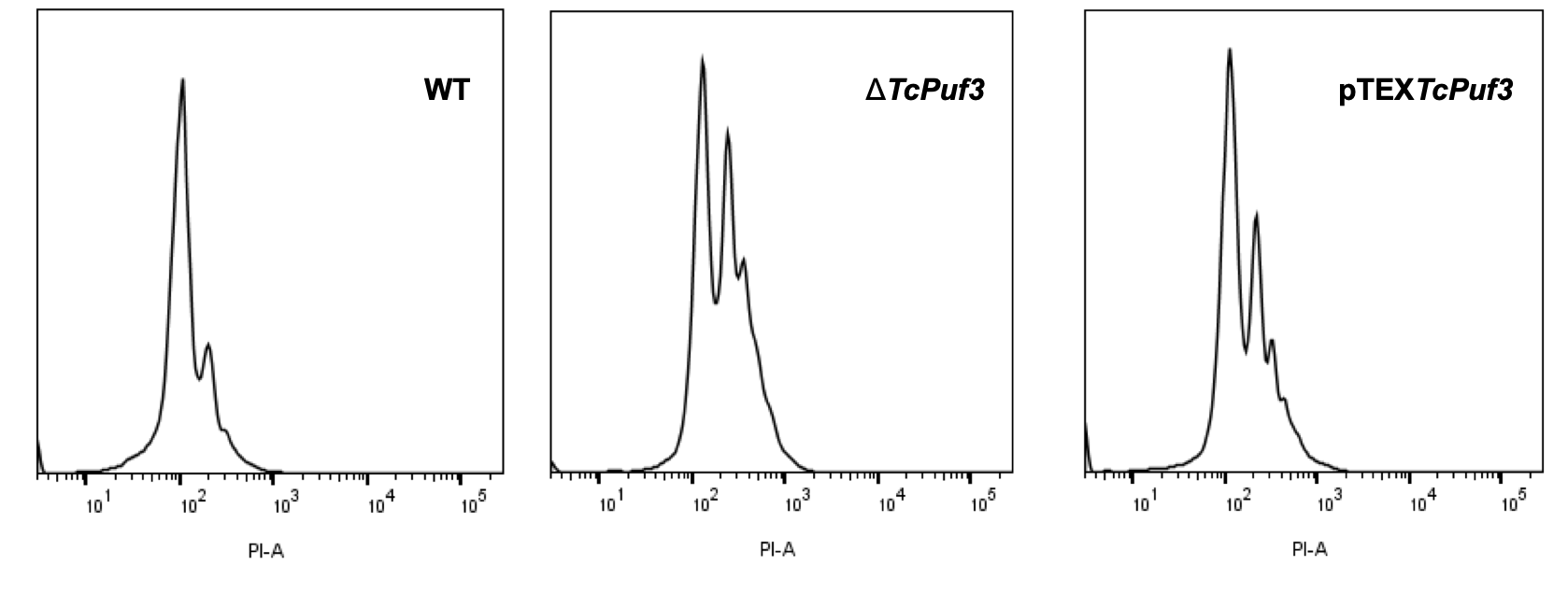


**Fig. S8**. **Flow cytometry analysis of cell cycle progression**. The WT, Δ*TcPuf3* and pTEX*TcPuf3* populations were synchronized with HU for 16 h and readings were performed on a BD LSRFortessa Cell Analyzer flow cytometer. The graphs showed the median fluorescence intensity of propidium iodide (PI).
